# How Stress Barriers and Fracture Toughness Heterogeneities Arrest Buoyant Hydraulic Fractures

**DOI:** 10.1007/s00603-024-03936-0

**Published:** 2024-05-20

**Authors:** Andreas Möri, Carlo Peruzzo, Dmitry Garagash, Brice Lecampion

**Affiliations:** 1 Institute of Civil Engineering, Geo-Energy Laboratory, Gaznat Chair on Geo-Energy, École polytechnique fédérale de Lausanne (EPFL), EPFL-ENAC-IIC-GEL Station 18, 1015 Lausanne, Vaud Switzerland; 2 Department of Civil and Resource Engineering, Dalhousie University, 5268 DaCosta Row, Halifax, NS B3H 4R2 Canada

**Keywords:** Fluid-driven fractures, Fluid-buoyancy, Heterogeneities

## Abstract

In our study, we investigated the impact of changes in Mode I fracture toughness and stress barriers on fully developed planar, buoyant hydraulic fractures assuming linear elastic hydraulic fracture mechanics. We present scaling-based arguments to predict the interaction type and use numerical simulations to validate our findings. Through a two-dimensional simplification, we estimate the lower limit for the fracture to feel a change in fracture toughness (so-called *immediate breakthrough*). Our simulations show that this approach only captures the order of magnitude of the toughness jump necessary for *immediate breakthrough* compared to the actual value due to three-dimensional solid effects, emphasizing their importance in such systems. We show that we can estimate the occurrence of *indefinite containment* at depth by considering that lateral spreading occurs at an approximately constant height. However, timing predictions in the case of a *transient containment* suffer from our simplified approach, which cannot model the injection history of the spreading constant height fracture. The same observations regarding *immediate breakthrough* and *indefinite containment* hold when considering stress barriers using pressure-scale-based arguments. Our study shows that the required toughness changes for fracture arrest are more significant than the observed values in the field. In contrast, stress barriers with a magnitude of around 1 MPa are generally sufficient to contain buoyant hydraulic fractures indefinitely. Stress barriers, in combination with other arrest mechanisms, are thus the most prominent mitigation factor of buoyant growth in industrially created hydraulic fractures.

## Highlights

- Derivation of a 2D simplification to decide how 3D planar buoyant hydraulic fractures interact with changes in the Mode I fracture toughness.
- Derivation of the scaling for the approximately constant height spreading along a Mode I fracture toughness jump of a buoyant hydraulic fracture.
- Derivation of the limiting volume and injection rate to contain a buoyant hydraulic fracture below a change in Mode I fracture toughness.
- First-order estimation of the limits for containment and interaction type of buoyant hydraulic fractures at a change in confining stress.
- Validation of the derivations and first-order estimations through fully coupled planar 3D simulations of buoyant hydraulic fractures.

## Introduction

Hydraulic fracturing treatments are commonly used in the petroleum and geothermal industry. Such treatments are designed to create opening mode (Mode I), tensile fractures by the injection of pressurized fluid at depth, with the ultimate goal to enhance the productivity of wells (Economides and Nolte 2000). Hydraulic fracturing is also used, albeit at a smaller scale, to determine the value of the minimum in-situ stress (Desroches and Thiercelin 1993). More recent applications in the subsurface also aim to use hydraulic fractures to store energy in the subsurface (Bunger et al. 2023; Hellström and Larson 2001). Hydraulic fractures are also observed naturally in the form of propagating magmatic intrusions like sills and dikes (Rivalta et al. 2015; Spence et al. 1987; Lister and Kerr 1991) and water crevassing in glaciers (Weertman 1971). In all these occurrences, hydraulic fractures propagate in a pre-compressed formation, perpendicular to the minimum in-situ stress (Detournay 2016).

**Fig. 1:**
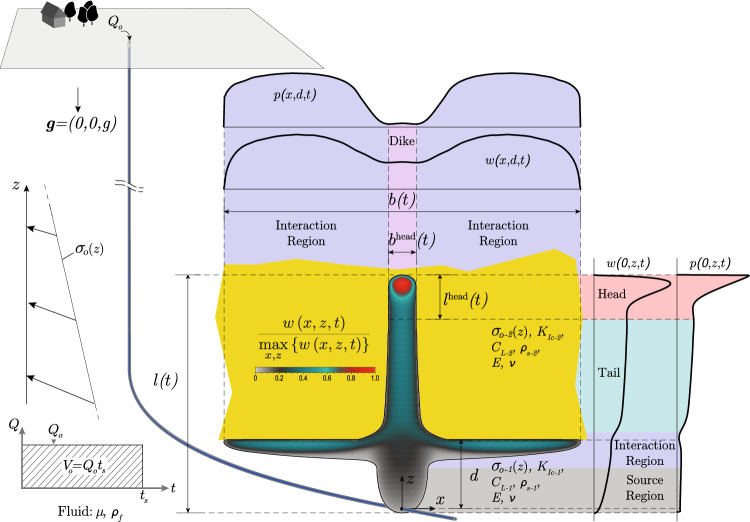
Schematic of the interaction between a buoyant hydraulic fracture and a change in the Mode I fracture toughness. Shown are the different characteristics and dimensions of the problem investigated and the resulting fracture. (Color figure online)

We focus hereafter on so-called “block injections”: fluid injected at a constant rate for a finite amount of time from a point source (see Fig. 1). The propagation phase of the resulting planar, radial (also called penny-shaped) hydraulic fracture is dependent on the interplay between the dominating energy dissipation mechanisms (fluid viscous dissipation versus fracture surface creation) and the amount of fluid leaking off through the fracture walls in the rock formation (storage versus leak-off) (Detournay 2016). In the case of radial hydraulic fractures, it has been shown by Savitski and Detournay (2002) that fractures transition from an early time regime where energy is predominantly dissipated in viscous flow (viscosity-dominated) to a late time regime where the energy to create new surfaces dominates (toughness-dominated). Both limits feature previously obtained self-similar solutions (see Spence and Sharp (1985) for the viscosity-dominated and Abé et al. (1976) for the toughness-dominated limits), and the transition between them is solely dependent on a dimensionless number (Savitski and Detournay 2002). The second balance between the volume of the fracture and the volume lost to the environment has been shown to feature a similar transition from an early-time storage-dominated (most of the fluid is still inside the fracture) to a late-time leak-off-dominated (most of the fluid has been lost to the formation) regime (Madyarova 2003). Similarly, the limiting regimes show self-similar solutions. The combined effect of all four limits can be captured by combining the two balances in a propagation diagram with four vertex solutions. The fracture’s exact evolution can be captured using a single dimensionless number, the so-called trajectory parameter (Detournay 2016). Only recently, the behavior of such radial fractures after the end of the injection, also called in the pulse regime, has undergone detailed evaluations. Möri and Lecampion (2021), focused on the characteristics and conditions when the fracture stops to propagate. They could notably show that fracture propagation after the end of the fluid injection is possible. For such fracture propagation, the fracture must be in the viscosity-storage-dominated regime when the injection stops. The development of appropriate asymptotes for the subsequent recession of a fracture has recently been derived by Peirce and Detournay (2022a), leading to the development of a late-time solution called the Sunset solution (Peirce and Detournay 2022b). In a coherent study including propagation, arrest, and recession of radial hydraulic fractures, Peirce (2022) demonstrated the appearance of the sunset solution.

The theoretical evolution of hydraulic fractures recalled above is based on the assumption of a homogeneous medium. However, most engineering applications occur in sedimentary basins, where formations generally show an intense layering with varying material properties between them. A similar variation is observed in the initial in-situ stresses. The study of the interaction with these layers has attracted much interest. It is worth noting that heterogeneities also exist at smaller scales, which can be homogenized (see, e.g., the field experiments of Jeffrey et al. (2009)). Such homogenization consists of lumping these small-scale heterogeneities into apparent macroscopic material properties. In this contribution, we consider heterogeneities at the scale of the fracture itself. The fracture can change its dynamics and/or propagation directions in relation to changes in material properties and confining stresses. Notably, the deviation along bedding planes, leading to so-called T-shape fractures, has been extensively studied (Bunger and Lecampion 2017; Xing 2018; Chen et al. 2015; Daneshy 1978, 2009; Chang et al. 2023). Here, we focus on the case where fracture propagation remains planar, but its shape is affected by heterogeneities or stress changes. The containment of hydraulic fractures between two layers has already been accounted for in the earliest models. The well-known PKN-model, named after its developers (Perkins and Kern 1961; Nordgren 1972), considered this case where the geological layering fixes the height of the fracture (sometimes also referred to as constant height or blade-like fractures). De Pater (2015) showed in a compilation of field observations that this fracture type is observed in various applications in hydrocarbon reservoirs. Such “blade-like” models of fracture geometry have then been extended and investigated at length in multiple settings up to today (Sarvaramini and Garagash 2015; Kovalyshen and Detournay 2009; Dontsov and Peirce 2016; Zolfaghari et al. 2017; Dontsov and Peirce 2015; Xing et al. 2017; Zia and Lecampion 2017; Dontsov 2022).

The discussion for the causes of fracture containment started in parallel with developing these models. Simonson et al. (1978) addressed the main factors leading to such confinement: stress or density contrasts between the layers or differences in elastic properties. Those factors and others have been extensively validated and studied numerically and theoretically (Cleary 1978; Daneshy 1978; Hanson et al. 1981; Warpinski et al. 1982b; van Eekelen 1982). Changes in fracture toughness only have been identified as a secondary effect for fracture containment because of their limited variability between layers (van Eekelen 1982; Gu and Siebrits 2008; Da Fies et al. 2022a, b). The possibility that the sole change in fracture toughness could contain three-dimensional (3D) planar fractures was demonstrated by various authors (Thiercelin et al. 1989; Li and Keer 1992; Ho and Suo 1993) and led to the development of the toughness-dominated PKN-solution, in contrast to the original viscosity-dominated, PKN-formulation (Sarvaramini and Garagash 2015). Linking the two regimes has only recently been done by Dontsov (2022), who showed that PKN fractures transition from an early-time toughness- to a late-time viscosity-dominated regime. Very recently, Peruzzo (2023) re-investigated the problem accounting for this transition and demonstrated the conditions for a “breakthrough” of the containing layers. However, the mechanism considered as most effective in containing hydraulic fractures is given by differences in the confining stress (Harrison et al. 1954; Simonson et al. 1978; Nolte and Smith 1981; Warpinski et al. 1982a, b; Bunger and Lecampion 2017). The commonly adopted theory as of today is based on Adachi et al. (2010) and accounts for the necessary penetration into the higher confinement stress layer through a so-called equilibrium height. The equilibrium height is a penetration depth of constant value into the higher stress layer, which allows the derivation of the governing equations of lateral expansion of such fractures.

In parallel with the study of hydraulic fracture containment in industrial applications, gravitational effects have been investigated in relation to magmatic intrusions. However, the same buoyant effects are notably applicable in anthropogenic hydraulic fractures because, in sedimentary basins, where most of the petroleum activity occurs, the minimum compressive stress is usually horizontal, leading to fracture growth in vertical planes aligned with the gravity vector (Hubbert and Willis 1957; Jaeger et al. 2007). Combining a significant fracture extent with the alignment with the gravity vector can lead to the emergence of so-called buoyant fractures. Such fractures have been studied since the pioneering work of Weertman (1971). Most investigations simplified the problem by considering an inviscid fluid to track the propagation path (Dahm 2000a, b; Davis et al. 2023), studying semi-infinite or finite two-dimensional (2D) configurations (Spence and Turcotte 1985, 1990; Spence et al. 1987; Lister 1990b; Lister and Kerr 1991; Roper and Lister 2005, 2007; Furst et al. 2023), using a pseudo-3D approach (Lister 1990a), were limited to the emergence of buoyant fractures without considering their growth (Davis et al. 2020; Salimzadeh et al. 2020), or assumed a late-time behavior according to a blade-like fracture of constant breadth (Garagash and Germanovich 2014, 2022). Only recently, the investigations of Möri and Lecampion (2022, 2023) have coherently clarified the typical three-dimensional behavior of hydraulic fractures emerging from a point source (constant fluid injection and finite volume) when transitioning from radial to buoyant propagation and their growth in the subsequent buoyant regime. Their results have shown that the entire propagation history of buoyant hydraulic fractures, in the absence of fluid-leak-off (e.g., in an impermeable media), is captured by only two dimensionless numbers. The first relates to the dimensionless number governing radial growth when buoyant forces become of order one. The second relates the total fluid volume released to a critical volume derived from the pioneering work of Weertman (1971).

Due to the analogy between rising magmatic intrusions and buoyant hydraulic fractures, the interaction of these fractures with changes in lithology has a strong practical interest. The main reason for this interest is to infer the possibility of an intrusion reaching the surface. The most commonly studied effects influencing the propagation of magmatic intrusion in this context are variations of elastic properties (see e.g., Fridleifsson 1977; Hyndman and Alt 1987; Rivalta et al. 2005; Kavanagh et al. 2006; Burchardt 2008; Gudmundsson 2011; Maccaferri et al. 2010; Furst et al. 2023) and density contrasts (see e.g., Lister 1990a, 1991; Lister and Kerr 1991; Muller et al. 2001; Watanabe et al. 2002; Pinel and Jaupart 2004; Taisne and Jaupart 2009; Taisne et al. 2011). Maccaferri et al. (2011) additionally investigate the influence of a weak interface (smaller energy release rate) between layers of different elastic compliance. Studies that mainly focused on the trajectory parameter have investigated the effect of topographical loads and other heterogeneous stress states (see, e.g., Johnson and Pollard 1973; Pollard and Johnson 1973; Gudmundsson and Marinoni 1999; Dahm 2000a; Menand et al. 2010; Menand 2011; Ferrante et al. 2022). The interaction with different fracture toughness values or a stress jump has only obtained limited interest. The authors are, however, aware of an experimental study conducted by Rivalta et al. (2005), which investigated the effect of toughness heterogeneities on propagating buoyant fractures in gelatine. These experiments of air-filled cracks show that in the limit of large-fracture toughness, 2D approximations work fairly well in predicting the shape of buoyant fractures. Generally, the previously mentioned studies are either experimental and most often out of the well-defined limits for buoyant propagation defined in (Möri and Lecampion 2023) or—if numerical—remain 2D approximations.

**Fig. 2:**
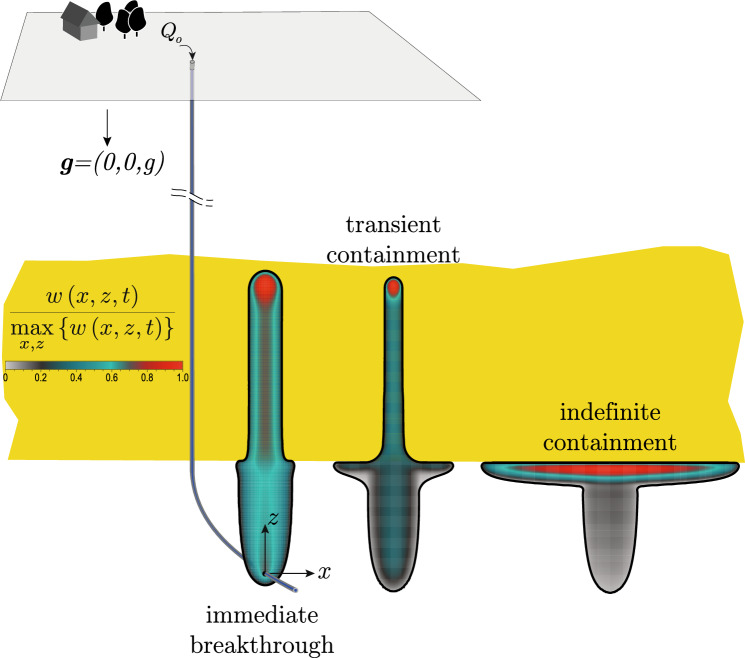
Possible outcomes of a buoyant hydraulic fracture interacting with a toughness jump. From left to right, we have *immediate breakthrough* (the effect of the toughness jump is negligible), *transient containment* (the fracture spreads out at the interface but ultimately forms a fracture growing into the upper layer), and *indefinite containment* (the fracture spreads out at the interface and never goes through). (Color figure online)

This study aims to combine the knowledge gained on buoyant hydraulic fractures with an understanding of the interaction of stress and toughness changes for planar hydraulic fractures. We thus want to investigate how buoyant hydraulic fractures in industrial applications interact with the layered nature of sedimentary basins. This study also aims to understand why magmatic intrusions regularly arise to full surface eruptions, whereas the same cannot be said about hydraulic fractures created by industrial injections. The study assumes linear elastic fracture mechanics in an elastic homogeneous, isotropic medium and perfectly planar hydraulic fractures. We account for a linear variation in the minimum compressive in-situ stress $\sigma _\textrm{o}$ (see Fig. 1) unless stated otherwise. The viscous fluid flow inside the thin fracture is considered a parallel plate flow. This result comes from the assumption of lubrication flow and a width-averaged continuity equation for incompressible fluids, leading to the applicability of the cubic law (Batchelor 1967). The fracturing fluid is assumed to be Newtonian, and we consider the elastic medium as impermeable. Finally, the propagation condition follows the linear elastic hydraulic fracture mechanics tip-asymptotes (see, e.g., Detournay (2016) for a review), ensuring that the stress intensity factor at propagating segments of the fronts equals the Mode I fracture toughness (see Sect. 2.1 for more details). The resulting equations are solved using the in-house developed, open-source boundary element solver PyFrac (Zia and Lecampion 2020). The code is a boundary element implementation of the implicit-level set algorithm (Peirce and Detournay 2008) and has been extensively tested. It has notably performed well for buoyancy-driven fractures (Möri and Lecampion 2022, 2023) and problems including heterogeneities (Peruzzo 2023). This paper thus consists of an extension of the work of Möri et al. (2023b). They have investigated similar cases of stress and toughness contrasts and identified three possible modes of interaction between the fracture and these property changes, visualized in Fig. 2. The most straightforward interaction is an *immediate breakthrough*. In this scenario, the fracture does not feel the effect of the change and grows directly into the upper layer without significant lateral growth. The other two limits are characterized by an arrest of the vertical growth and a subsequent spreading along the interface (see Fig. 2). The *transient containment* shows a so-called breakthrough sometimes after the fracture has reached the change in property/stress. Breakthrough is defined as a self-sustained buoyant fracture being able to develop in the upper layer. The third category of *indefinite containment* is a fracture which laterally spreads along the change of property/stress without ever generating a self-sustained buoyant fracture in the upper layer. Interestingly, the physical experiments performed in toughness-dominated conditions of Rivalta et al. (2005) have also shown these exact outputs for the values they considered.

In the remainder of this article, we will give a short description of the mathematical formulation (Sect. 2.1) followed by a discussion of past developments considering buoyant hydraulic fractures in a homogeneous medium (Sect. 2.2). We then introduce some representative cases studied in this contribution (Sect. 2.3. Using these representative values we then address changes in the value of fracture toughness (Sect. 3) and develop scaling-based arguments for an *immediate breakthrough* (Sect. 3.1), and the limit between *transient* and *indefinite containment* (Sect. 3.2), which we validate through numerical simulations in Sects. 3.1.1 and 3.2.1. The same principles are then applied to a change in background stress (also called stress barriers—Sect. 4). We conclude the paper with a discussion of other possible arrest mechanisms and their combination (Sect. 5) before highlighting the main conclusions of this article (Sect. 6).

## Methods

### Mathematical Formulation

This contribution focuses on the case of pure opening mode (Mode I) fractures remaining planar during all their propagation history. As shown in Fig. 1, we consider fluid injections from a point source at a constant rate $Q_\textrm{o}$ for a finite amount of time $t_\textrm{s}$. Consequently, $V_\textrm{o} = Q_\textrm{o} t_\textrm{s}$ is the total volume injected. The propagation plane is vertical, so the gravity vector $\textbf{g}$ is aligned with it. Thanks to these assumptions, the quasi-static balance of momentum in a linear elastic medium can be reduced to the following boundary integral equation over the fracture surface $\mathcal {A}(t)$ (Crouch and Starfield 1983; Hills et al. 1996)

1 $$\begin{aligned}{} & {} p\left( x,z,t\right) =p_\textrm{f}\left( x,z,t\right) -\sigma _\textrm{o}\left( x,z\right) \nonumber \\{} & {} \quad =-\frac{E^{\prime }}{8\pi }\int _{\mathcal {A}(t)}\frac{w\left( x^{\prime },z^{\prime },t\right) }{\left[ \left( x^{\prime }-x\right) ^{2}+\left( z^{\prime }-z\right) ^{2}\right] ^{3/2}}\text {d} {x^{\prime }}\text {d} z^{\prime }. \end{aligned}$$

where $E^{\prime } = E/\left( 1-\nu ^2\right)$ is the materials plane strain Modulus with *E* its Young’s Modulus and $\nu$ its Poisson’s ratio, $p\left( x,z,t\right) =p_\textrm{f}\left( x,z,t\right) -\sigma _\textrm{o}\left( x,z\right)$ the net pressure in the fracture with $p_\textrm{f}\left( x,z,t\right)$ the fluid pressure and $\sigma _\textrm{o}\left( x,z\right)$ the confining minimum horizontal stress, and $w\left( x,z,t\right)$ the fracture opening. The material is further considered impermeable, and we apply the thin film lubrication approximation for an incompressible fluid to obtain the volume balance in the fracture (Batchelor 1967)

2 $$\begin{aligned} \frac{\partial w\left( x,z,t\right) }{\partial t}+\nabla \cdot \left( w\left( x,z,t\right) \textbf{v}_\textrm{f}\left( x,z,t\right) \right) =\delta (x)\delta (z)Q_\textrm{o}(t), \end{aligned}$$

where $\textbf{v}_\textrm{f}\left( x,z\right)$ is the width averaged fluid velocity. We further assume laminar flow and Newtonian fluid rheology to obtain the flow in the fracture according to Poiseuille’s law

3 $$\begin{aligned}{} & {} \textbf{q}\left( x,z,t\right) =w\left( x,z,t\right) \textbf{v}_\textrm{f}\left( x,z,t\right) \nonumber \\{} & {} \quad =-\frac{w\left( x,z,t\right) ^{3}}{\mu ^{\prime }}\left( \mathbf {\nabla }p_\textrm{f}\left( x,z,t\right) -\rho _\textrm{f}\textbf{g}\right) , \end{aligned}$$

where $\mu ^{\prime } = 12\mu$ is the adapted fracturing fluids viscosity with $\mu$ its viscosity and $\rho _\textrm{f}$ its density. Using again the fluid net pressure ${\displaystyle p\left( x,z,t\right) }$ we obtain

4 $$\begin{aligned} \textbf{q}\left( x,z,t\right) =-\frac{w\left( x,z,t\right) ^{3}}{\mu ^{\prime }}\left( \mathbf {\nabla }p\left( x,z,t\right) +\varDelta \gamma \frac{\textbf{g}}{\left| \textbf{g}\right| }\right) . \end{aligned}$$

where the fluid-solid system is subjected to the constant buoyancy

5 $$\begin{aligned} \varDelta \gamma = \left( \alpha \left( \rho _\textrm{s} - \rho _\textrm{f}\right) + \rho _\textrm{F} - \rho _\textrm{f}\right) g \approx \alpha \left( \rho _\textrm{s} - \rho _\textrm{f}\right) g. \end{aligned}$$

In Eq. (5), we have included the classical lateral earth pressure coefficient in rocks $\alpha = \nu /\left( 1-\nu \right)$ to calculate the system’s buoyancy (Jaeger et al. 2007). We further assumed that the formation fluid has the density of water $\rho _\textrm{F} = \rho _w$ and is thus approximately equal to the density of the injection fluid $\rho _\textrm{f} \approx \rho _w$, that the gradient of the background stress is lithostatic (with $\rho _\textrm{s}$ the density of the solid), and that the background pore fluid pressure gradient is hydrostatic (Heidbach et al. 2018; Cornet 2015; Jaeger et al. 2007). In this study, we further consider that fractures are deep within the Earth’s crust such that the high confining stresses lead to a negligible fluid lag (Garagash and Detournay 2000; Lecampion and Detournay 2007; Detournay 2016). Consequently, the boundary conditions are zero fracture width ${\displaystyle \left( w\left( x_\textrm{c},z_\textrm{c}\right) =0\right) }$ and a zero normal fluid flux ${\displaystyle \left( \textbf{q}\left( x_\textrm{c},z_\textrm{c}\right) =0\right) }$ at the fracture front (Detournay and Peirce 2014).

In the framework of linear elastic hydraulic fracture mechanics, we assume propagation is in quasi-static equilibrium such that the propagation condition becomes

6 $$\begin{aligned} \left( K_\textrm{I}\left( x_\textrm{c},z_\textrm{c}\right) -K_\textrm{Ic}\right) v_\textrm{c}\left( x_\textrm{c},z_\textrm{c}\right) =0\qquad v_\textrm{c}\left( x_\textrm{c},z_\textrm{c}\right) \ge 0\qquad K_\textrm{I}\left( x_\textrm{c},z_\textrm{c}\right) \le K_\textrm{Ic}, \end{aligned}$$

for all ${\displaystyle \left( x_\textrm{c},z_\textrm{c}\right) \in {\displaystyle \mathcal {C}\left( t\right) }}$, meaning for all points on the fracture front. In this equation, ${\displaystyle v_\textrm{c}\left( x_\textrm{c},z_\textrm{c}\right) }$ is the local fracture velocity normal to the front, $K_\textrm{I}\left( x_\textrm{c},z_\textrm{c}\right)$ is the local stress intensity factor, and $K_\textrm{Ic}$ the fracture toughness.

### Scalings of Buoyant Hydraulic Fractures

Under the assumptions lined out in Sect. 2.1, Möri and Lecampion (2022, 2023) have demonstrated that the entire propagation history of a buoyancy-driven hydraulic fracture in homogeneous stress and material conditions depends on only two dimensionless numbers. The first dimensionless number is the dimensionless viscosity of a radial hydraulic fracture at the moment when buoyancy becomes of order one (e.g., the fracture size approaches the buoyancy length scale $\ell _\textrm{b}$)

7 $$\begin{aligned} \mathcal {M}_{\widehat{k}} = \mu ^{\prime }\frac{Q_\textrm{o} E^{\prime 3} \varDelta \gamma ^{2/3}}{K_\textrm{Ic}^{14/3}}. \end{aligned}$$

$\mathcal {M}_{\widehat{k}}$ describes the dominant energy dissipation mechanisms when the fracture transitions from the axisymmetric radial to the unidirectional buoyant growth. Möri and Lecampion (2022) have shown that it entirely governs the propagation history as long as the fluid injection takes place. The larger $\mathcal {M}_{\widehat{k}}$ gets, the more lateral fracture growth is possible thanks to additional energy dissipation by viscous flow. This large viscous energy drop leads to higher pressures, such that a laterally non-stabilized propagation regime with sub-linear fracture growth is possible for a long time. A second and more important interpretation of $\mathcal {M}_{\widehat{k}}$ is that it characterizes the amount of fluid a buoyant hydraulic fracture can store in its head (see Fig. 1 for a definition of the fracture head). This increase in head volume leads to a higher accumulation of elastic energy, which is necessary to overcome the additional energy required by the viscous flow. The combined effect of changing values of $\mathcal {M}_{\widehat{k}}$ on fracture velocity and head volume directly governs how the buoyant fracture interacts with a change in fracture toughness or confining stress.

When the fluid injection is finite, a second dimensionless number must be considered to quantify the propagation history of buoyant hydraulic fractures. Möri and Lecampion (2023) decided to use the dimensionless buoyancy of the radial, toughness-dominated regime (*K*-regime) at the moment when the injection stops

8 $$\begin{aligned} \mathcal {B}_{ks} = \varDelta \gamma \frac{V_\textrm{o}^{3/5} E^{\prime 3/5}}{K_\textrm{Ic}^{8/5}}. \end{aligned}$$

The physical interpretation of $\mathcal {B}_{ks}$ is that it measures the total amount of fluid injected $V_\textrm{o}$ compared to the minimum volume necessary for buoyant propagation to occur in the first place. This volume has been identified by various contributors (Davis et al. 2020; Salimzadeh et al. 2020; Garagash and Germanovich 2022; Möri and Lecampion 2023). It is the equivalent volume of radial, hydrostatically loaded hydraulic fractures with a maximum stress intensity factor equal to the Mode I fracture toughness at one extremity and zero at the other. Once the injection has stopped, the buoyant fracture head will always shrink during its ascent until it reaches that minimum volume. For small values of $\mathcal {M}_{\widehat{k}}$ (toughness-dominated buoyant fractures $\widehat{K}$-regime), the head volume is already equivalent to the limiting volume. In all other cases, the head volume shrinks until it reaches this volume. The toughness-dominated buoyant finite volume regime ($\widehat{K}^{\left[ V\right] }$-regime) is thus the late-time solution of all finite volume buoyant hydraulic fractures. Due to these links, the two numbers $\mathcal {B}_{ks}$ (8) and $\mathcal {M}_{\widehat{k}}$ (7) can be used to describe the propagation history of these fractures entirely. The resulting parametric space is shown in figure 2 of Möri and Lecampion (2023). The parametric space and table 1 of Möri and Lecampion (2023) quantify the limiting regimes a fracture will encounter during its propagation and can hence give at which time the respective regime will be dominant.

In this study, we investigate the interaction with heterogeneities. These interactions have been shown to strongly depend on the fracture’s dominating energy dissipation mechanism at the moment when the buoyant fracture reaches the change in property/stress (Peruzzo 2023; Möri et al. 2023b). For the problem sketched in Fig. 1, this means when the upper fracture tip reaches the interface between two properties. We thus require an additional component to study the interaction: the time of contact or, alternatively, the distance between the injection point and this interface. We decide herein to choose the latter, a quantity easier to grasp, which can be related to the time using the buoyant hydraulic fracture scalings developed in Möri and Lecampion (2022, 2023). We thus introduce a third dimensionless number, the dimensionless distance

9 $$\begin{aligned} \mathcal {D} = d\frac{\varDelta \gamma ^{2/3}}{K_\textrm{Ic}^{2/3}}. \end{aligned}$$

**Fig. 3:**
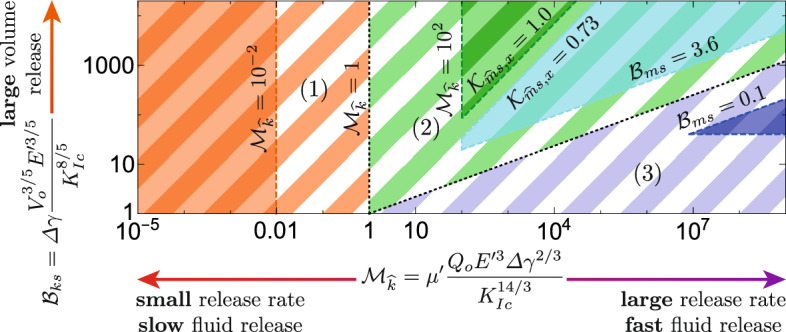
Parametric space of buoyant hydraulic fractures. Indicated are related dimensionless numbers and the regions 3–6 from Möri and Lecampion (2023) (full-colored regions). In dashed, we indicate three regions (1–3) for which we differentiate the fracture regimes when interacting with a change of property in Fig. 4.(Color figure online)

**Fig. 4:**
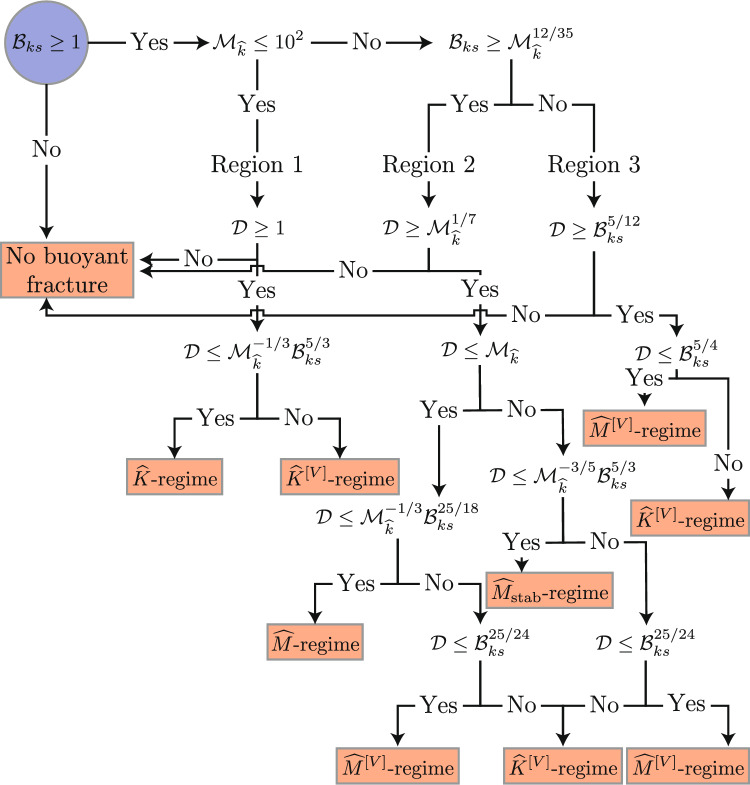
Regime of the fracture when reaching the change of properties/stress. Start in the left top corner (blue circle) and then follow the different conditions until you reach a red square marking the regime of interaction observed for the given parameter combination. (Color figure online)

This dimensionless distance $\mathcal {D}$ is the ratio between the physical initial distance *d* between the injection point and the interface and the toughness buoyancy length scale $\ell _\textrm{b} = K_\textrm{Ic}^{2/3} / \varDelta \gamma ^{2/3}$ (Weertman 1971; Lister and Kerr 1991). It is also the length scale attached to the limiting volume and, thus, the size of the fracture head in the late-time solution. We reproduce a slightly adapted version of figure 2 of Möri and Lecampion (2023) in Fig. 3, which together with Fig. 4 indicates in which state the buoyant hydraulic fracture will be when it encounters the change.

We note that Fig. 3 only contains the part relevant for fractures which are buoyant $\mathcal {B}_{ks} \ge 1$. According to table 1 of Möri and Lecampion (2023), the three regions reproduced indicate propagation histories containing all possible buoyant regimes. We perform at this stage a brief recall of the respective characteristics of buoyant hydraulic fractures in these limiting regimes. Note that the limits for the application given in the paragraph titles are approximations, and more in-depth analysis according to the work presented in Möri and Lecampion (2022, 2023) would be necessary to definitively distinguish the respective limits.

$\widehat{K}$*-regime* $\mathcal {M}_{\widehat{k}} \ll 1$ *and* $t < t_\textrm{s}$ The toughness-dominated regime in an ongoing injection case shows the classical "blade-like" fracture with a head of constant volume and shape and a tail of uniform breadth with constant opening (see Sect. 4 of (Möri and Lecampion 2022) for more details). For a constant injection rate, such a buoyant fracture propagates at a constant velocity in the direction of the buoyant force. If the change in property/stress is reached in this regime, the distance *d* does not influence the interaction type as the velocity is constant.

$\widehat{M}$*-regime and *$\widehat{M}_{stab}$*-regime* $\mathcal {M}_{\widehat{k}} \gg 1$, $t < t_\textrm{s}$ The viscosity-dominated regime without lateral stabilization $\widehat{M}$-regime in an ongoing injection case shows fractures with an inverse cudgel size that continue to grow laterally (e.g., no fixed breadth). Their lateral growth leads to a sub-linear vertical growth and a reduction of the size of the fracture head (see Sect. 5.1 of (Möri and Lecampion 2022) for more details). The distance between the injection point and the interface thus affects, through a change in the fracture velocity and the head volume, how the interaction between the fracture and the change will be. Lateral growth is bounded if the toughness is finite, and the so-called stabilized viscosity-dominated buoyant regime emerges $\widehat{M}_\textrm{stab}$-regime. Here, the head becomes of constant volume and shape again, and propagation returns to a linear growth in time. The constant velocity thus again leads to independence of the interaction type on the distance *d* (see Sect. 6 of (Möri and Lecampion 2022) for more details).

$\widehat{K}^{\left[ V\right] }$*-regime* $\mathcal {M}_{\widehat{k}} \ll 1$, $t > t_\textrm{s}$: The fractures in this regime are similar to the $\widehat{K}$-regime "blade-like" with the exact same head volume and shape. The only difference is that the injection has stopped, and fractures grow sub-linearly by depleting the fluid volume from the tail (see Sect. 5.1 of Möri and Lecampion (2023) for more details). This sub-linear creates again a dependence of the interaction with the distance *d*.

$\widehat{M}^{\left[ V\right] }$*-regime* $\mathcal {M}_{\widehat{k}} \gg 1$, $t > t_\textrm{s}$: Buoyant fractures in this regime show a head with a volume exceeding the one of toughness-dominated fractures and a non-uniform breadth of the tail. During their ongoing propagation forced by a depletion of the tail, the head of these fractures shrinks to reach the minimum size given by the head volume of the $\widehat{K}^{\left[ V\right] }$-regime and $\widehat{K}$-regime. The propagation in between these limits can be faster than at the end but remains sub-linear in all conditions (see Sect. 5.2 of Möri and Lecampion (2023) for more details). Finally, the distance *d* will always be an important factor for such fractures in accessing the interaction with the property/stress change.

Figure 4 shows the variety of possible states a fracture could be in when encountering a change of the rock property or in the background stress. From this variety of possible interactions, we can conclude that exploiting all these different limits in detail using fully planar 3D simulations would be exhaustive. We thus identify two representative combinations of parameters for industrial applications hereafter and try to investigate their possible interaction with a stress and fracture toughness jump. We will investigate the two cases numerically and through scaling arguments.

### Representative Cases

We have compiled a study of multiple shale formations focusing on those in the United States. The estimation of the mechanical properties of the formation is primarily taken from  Dobson and Houseworth (2013) and other authors (Schwartz et al. 2019; Kong et al. 2023; Zhou et al. 2023; Jin et al. 2018; Jiang et al. 2018). We assume a slickwater injection into horizontal wells. In particular, we focus on a single-stage injection and do not consider the addition of proppant in our model. The average rock density is taken as $\rho _\textrm{s} = 2485$ kg/m^3^, and we consider a Poisson’s coefficient of $\nu = 0.2$. Including these values into (5), the values for $\Delta \gamma$ reported in Table 1 are obtained.

**Table 1:** Two limiting cases of material properties for a weak and a strong formation

	*E*	$\nu$	$K_\textrm{Ic}$	$\mu$	$\varDelta \gamma$	$Q_\textrm{o}$		$V_\textrm{o}$	
	GPa		MPa m^1/2^	Pa s	Pa / m	m^3^/s	BPM	m^3^	Gals
Weak	7.50	0.2	0.25	0.005	3643	0.12	60	1514	$4\times 10^5$
Strong	18.0	0.2	2.00	0.005	3643	0.24	120	5678	$1.5\times 10^6$

	$\mathcal {M}_{\widehat{k}}$	$\mathcal {B}_{ks}$	$\ell _\textrm{b}$		Immediate breakthrough	Indef. containment
			m	ft	$K_{{\text {Ic-2}}}/K_{{\text {Ic-1}}}\vert _{d = 175}$	$K_{{\text {Ic-2}}}/K_{{\text {Ic-1}}}\vert _{d = 175}$
Weak	$5.21 \times 10^4$	587	16.8	55.1	10.86	18.7
Strong	87.9	78.7	67.1	220	2.89	4.97

From the compiled rock formation data, we define two scenarios with the respective upper and lower limits of estimated injections. We consider a “strong” formation (large fracture toughness and Young’s modulus) in which we inject a large volume at a high rate and a “weak” formation (small fracture toughness and Young’s modulus) in which we inject a small volume at a low rate. The resulting values of the dimensionless numbers $\mathcal {M}_{\widehat{k}}$ (7) and $\mathcal {B}_{ks}$ (8) and the respective values of the parameters can be found in Table 1.

The first observation of the compiled data and the dimensionless coefficients calculated is that both injections should theoretically become buoyant and ascend (e.g., $\mathcal {B}_{ks} \ge 1$). In Table 1, we further list the buoyancy length scale. The buoyancy length scale is crucial to estimate the dimensionless coefficient $\mathcal {D}$ (9), which we need to estimate the fractures’s regime when it encounters the change. Our compilation of data with a focus on the work of Dobson and Houseworth (2013) has given us an average maximum height of rock formations of about 300–350 m (1000–1500 ft) and a minimum height that can be as low as 50 m (about 150 ft). Neglecting heterogeneities and bedding planes inside the rock formation, we can estimate that the major changes in properties occur at the boundary of these formations. Comparing the 50 m of the “small” formations to the buoyancy length scale, we obtain a dimensionless distance of $\mathcal {D}_{\text {50 m}} = 2.98$ for the weak and 0.75 for the strong formation. From Fig. 3, we estimate that both sets fall into the second region and thus require the condition that $\mathcal {D} \ge \mathcal {M}_{\widehat{k}}^{1/7}$ to be buoyant when encountering the limit. For both parameter combinations, this condition is not met. We can note here that three combinations of scenarios are possible. First, the fracture can encounter the heterogeneity when buoyant forces are negligible $\ell _\textrm{b} \gg d$. Such cases have been extensively studied, and their main conclusions state that the most effective ways to contain such fractures are changes in the confining stress and/or differences in elastic properties (see the Sect. 1 for references). Second, it might be that the distance between the injection point and the heterogeneity and buoyancy are on the same order $\ell _\textrm{b} \sim d$. This is the case here; no clear distinction between limiting regimes can be made in this limit, and their investigation must most likely follow a numerical approach. Third, a fully established buoyant fracture encounters the change $\ell _\textrm{b} \ll d$. This third category of fractures is what we are interested in this contribution. We can observe this case when taking the upper limit of the rock formation height. More precisely, we assume here that the fluid injection occurs at the centre of the layer such that we have $d = 175$ m leading to a dimensionless distance of $\mathcal {D}_{\text {175 m}} = 10.4$ for the weak and 2.61 for the strong formation. Following now the evaluation path described in Fig. 4, we obtain that both combinations lead to a buoyant hydraulic fracture encountering the change in the viscosity-dominated buoyant injection regime ($\widehat{M}$-regime).

## Change in Fracture Toughness

We first investigate the effect of a sudden change in fracture toughness on fracture propagation. For this type of heterogeneity, we define the limit of *immediate breakthrough* as validated if the fracture height growth does not stop at any moment $\partial \ell \left( t\right) /\partial t \ne 0, \forall t$. For a *transient containment* along a jump in fracture toughness, the spreading leads to an increase of the fracture opening at the point of contact such that the stress-intensity factor there increases up to the resistance of the higher toughness layer. Once this point is reached, the fracture breaks through into the upper layer, and grows there as a buoyant fracture. The discussion on a similar effect, when PKN fractures transition from their early-time toughness-dominated behavior to the late-time viscosity-dominated behavior, has been investigated in Dontsov (2022); Peruzzo (2023). On the other hand, once the fluid injection ends, the opening and stress-intensity factor at the point of contact can no longer increase. If the fracture has not yet broken through when the injection stops, the fracture will be *indefinitely contained*. In their study with different parameter sets at an arbitrary distance of $d = 250$ m ($d = 820$ ft), Möri et al. (2023b) have observed all three regimes as a function of typical toughness jumps between $K_{{\text {Ic-2}}}/K_{{\text {Ic-1}}} = 2$–5. Hereafter, we are interested in distinguishing the limits between the three regimes presented more analytically.

### Limit of Immediate Breakthrough

We follow hereafter the procedure outlined in Peruzzo (2023) to derive the necessary limit for an immediate breakthrough of the fracture. In chapter 5 of his work, Peruzzo (2023) uses the fact that the hydraulic fracture can be assumed as a plane-strain semi-infinite, steadily moving hydraulic fracture at the propagating edge. This fundamental assumption, inherent to the implicit level-set scheme (Peirce and Detournay 2008; Zia and Lecampion 2020), allows the author to perform a local energy balance for a semi-infinite fracture (see equation (5.8) of Peruzzo (2023)). His derivations neglect the presence of gravity. Incorporating a gravity effect as outlined in Fig. 1 would lead to an additional term in the power balance of the semi-infinite steadily moving fracture, leading to

10 $$\begin{aligned}{} & {} \mu ^{\prime }V^2{\int _\textrm{o}}^h \frac{1}{\widehat{w}}\text {d}\widehat{s} + G \times V + V\frac{1}{2}{\int _\textrm{o}}^h \frac{\text {d}}{\text {d}\widehat{s}}\left( \widehat{w}\widehat{p}\right) \text {d}\widehat{s}\nonumber \\{} & {} \quad = - V\Delta \gamma {\int _\textrm{o}}^h \widehat{w}\text {d}\widehat{s} + V\widehat{w}\left( h\right) \widehat{p}\left( h\right) . \end{aligned}$$

**Fig. 5:**
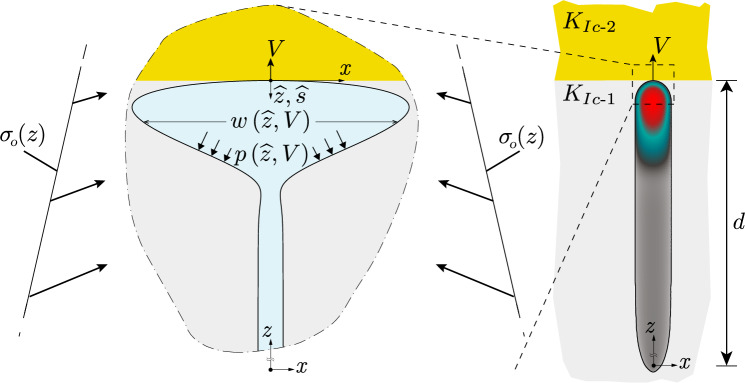
Sketch of a semi-infinite, buoyant hydraulic fracture encountering a change in fracture toughness. All other parameters remain the same. (Color figure online)

In Eq. 10, we have used the $\widehat{\cdot }$ notation to indicate that all quantities are considered in a moving frame at a constant velocity *V*. $\widehat{w}$ and $\widehat{p} = \widehat{p}_\textrm{f} - \sigma _\textrm{o}$ are the opening and net pressure, with $\widehat{p}_\textrm{f}$ the fluid pressure, in the moving coordinate system based on the fracture tip and *h* is a characteristic height over which the integrals are to be evaluated. We further use *G* for the elastic fracture mechanics energy release rate (Rice 1972). It has been pointed out by Garagash (2009); Garagash et al. (2011) that at the fracture tip we have the condition $\widehat{w}\left( 0\right) \widehat{p}\left( 0\right) = 0$. Applying this condition to Eq. 10, dividing by *V*, and applying the resulting equation to the propagation condition (6) (e.g., the energy release rate equals the critical energy release rate $G = G_\textrm{c} = K_\textrm{Ic}^2/E^{\prime }$) we obtain

11 $$\begin{aligned} - \Delta \gamma {\int _\textrm{o}}^h \widehat{w}\text {d}\widehat{s} -\mu ^{\prime }V{\int _\textrm{o}}^h \frac{1}{\widehat{w}}\text {d}\widehat{s} + \frac{1}{2}\widehat{w}\left( h\right) \widehat{p}\left( h\right) - G_\textrm{c} = 0, \end{aligned}$$

which holds for the condition that $V \ge 0$. We follow the procedure of Peruzzo (2023) again and consider the fracture as a semi-infinite steadily moving fracture when it encounters a change in fracture toughness (see Fig. 5). We now distinguish between a state just before the fracture touches the interface (state $\cdot ^{\left[ -\right] }$) and one just after the contact (state $\cdot ^{\left[ +\right] }$). Adopting the assumption that the change in energy entering the system at the injection point $\widehat{z} = d$, the change in the external elastic energy $\widehat{w}\widehat{p}$, and the change in the potential energy related to buoyancy between the two states are negligible

12 $$\begin{aligned}{} & {} \frac{1}{2}\widehat{w}^{\left[ -\right] }\left( h\right) \widehat{p}^{\left[ -\right] }\left( h\right) - \Delta \gamma {\int _\textrm{o}}^h \widehat{w}^{\left[ -\right] }d \nonumber \\{} & {} \quad = - \Delta \gamma {\int _\textrm{o}}^h \widehat{w}^{\left[ +\right] }d + \frac{1}{2}\widehat{w}^{\left[ +\right] }\left( h\right) \widehat{p}^{\left[ +\right] }\left( h\right) , \, \text {at: } t = t_d, \end{aligned}$$

where $t_d$ is the time the fracture tip reaches the interface at a distance *d*. The combination of Eqs. (11) and (12) allows us to define the limit where the fracture velocity after touching the interface becomes zero $V^{\left[ +\right] } = 0$. We define this case as an *immediate breakthrough* because the fracture never stops growing (Peruzzo 2023). Using the total energy dissipated (Eq. (11) for states $\cdot ^{\left[ -\right] }$ and $\cdot ^{\left[ +\right] }$) with the assumption of non-penetration into the higher toughness layer (e.g., $V^{\left[ +\right] } = 0$) we can obtain the following relation between fracture energies

13 $$\begin{aligned} G_{\text {c-2}} \ge G_{\text {c-1}} + \mu ^{\prime }V^{\left[ -\right] }{\int _\textrm{o}}^h \frac{1}{\widehat{w}^{\left[ -\right] }}\text {d}\widehat{s}. \end{aligned}$$

For co-planar fractures in an elastically homogenous medium, we can express this equation in function of the respective fracture toughness

14 $$\begin{aligned} \left( \frac{K_{{\text {Ic-2}}}}{K_{{\text {Ic-1}}}}\right) ^2 = 1 + \frac{\mu ^{\prime }V^{\left[ -\right] }E^\prime }{K_{{\text {Ic-1}}}^2} {\int _\textrm{o}}^h \frac{1}{\widehat{w}^{\left[ -\right] }}\text {d}\widehat{s}. \end{aligned}$$

In Peruzzo (2023), a similar equation to (14) (see his equation (5.16)) is cast into a non-dimensional form using the typical scales of semi-infinite hydraulic fractures shown in Garagash (2009); Garagash et al. (2011). We follow a similar path using semi-infinite buoyant hydraulic fracture scales slightly adapted from the supplemental material of Möri and Lecampion (2022)

15 $$\begin{aligned} \ell _\textrm{b}^{\text {2D}}=\frac{E^{\prime 1/2}Q_{2D}^{1/6}\mu ^{\prime 1/6}}{\varDelta \gamma ^{2/3}},\quad w_\textrm{b}^{\text {2D}}=\frac{Q_{2D}^{1/3}\mu ^{\prime 1/3}}{\varDelta \gamma ^{1/3}}, \quad \text {with } Q_{2D}=Vw^{\text {3D}}_{*}. \end{aligned}$$

In Eq. (15), $w^{\text {3D}}_{*}$ is the 3D scale of the tail at the moment when the switch between two- and three-dimensions is performed. Roper and Lister (2005, 2007) have shown in their work that a dimensionless toughness can characterize such a semi-infinite fracture. They presented a limiting solution in the toughness-dominated regime and solved it numerically for finite toughness cases. We use here a slightly different definition of the dimensionless tip-toughness as

16 $$\begin{aligned} \kappa = \frac{K_\textrm{Ic}}{E^{\prime 3/4}\mu ^{\prime 1/4}Q_{2D}^{1/4}}. \end{aligned}$$

We will use the same solver as presented in the supplemental material of Möri and Lecampion (2022), which has been validated there against the limiting solutions presented by Roper and Lister (2005, 2007) and a zero-toughness solution shown in Lister (1990b). Using the scaling of Eqs. (15), (16), and $\mathcal {H} = h/\ell _\textrm{b}^{\text {2D}}$, we can obtain a dimensionless equation giving the maximum change in fracture toughness leading to an *immediate breakthrough*

17 $$\begin{aligned} \left( \frac{K_{{\text {Ic-2}}}}{K_{{\text {Ic-1}}}}\right) ^2 = 1 + \kappa ^{-2} \frac{V^{\left[ -\right] }\mu ^{\prime 1/3}}{Q_{2D}^{2/3}\varDelta \gamma ^{1/3}} {\int _\textrm{o}}^\mathcal {H} \frac{1}{\widehat{\Omega }^{\left[ -\right] }}\text {d}\widehat{\xi }. \end{aligned}$$

In Eq. (17), we have used the following two scales $\widehat{\Omega } = \widehat{w}/w_\textrm{b}^{\text {2D}}$ and $\widehat{\xi } = \widehat{s}/\ell _\textrm{b}^{\text {2D}}$. It is possible to show that the term $V^{\left[ -\right] }\mu ^{\prime 1/3}/\left( Q_{2D}^{2/3}\varDelta \gamma ^{1/3}\right) = 1$ is valid in all scalings of buoyant hydraulic fractures, such that we can further simplify Eq. (17) to

18 $$\begin{aligned} \left( \frac{K_{{\text {Ic-2}}}}{K_{{\text {Ic-1}}}}\right) ^2 = 1 + \kappa ^{-2} {\int _\textrm{o}}^\mathcal {H} \frac{1}{\widehat{\Omega }^{\left[ -\right] }} \text {d}\widehat{\xi }. \end{aligned}$$

This equation allows us to obtain the limiting solution in function of the corresponding dimensionless tip-toughness by solving numerically for the corresponding opening. As Möri and Lecampion (2022) have shown that the complete 3D solution approaches the 2D solution relatively well in the toughness-dominated limit (see their figure 3) and very well in the viscosity-dominated limit (see their figure 7), it is possible to derive the limit of immediate breakthrough from Eq. (18) when a scaling based expression of $\kappa$ is possible.

#### Results for the Representative Cases

For the cases presented in Table 1, both fractures encounter the toughness jump in the viscosity-dominated buoyant injection regime ($\widehat{M}$-regime). For this regime we can derive the value of $\kappa$ as a function of the dimensionless viscosity $\mathcal {M}_{\widehat{k}}$ (7) and the dimensionless distance $\mathcal {D}$ (9) (see Appendix 1 for its value in other regimes)

19 $$\begin{aligned} \kappa _{\widehat{m}} = \mathcal {D}^{3/40}\mathcal {M}_{\widehat{k}}^{-9/40}. \end{aligned}$$

Note that we have not considered any pre-factor when performing the derivation of $\kappa _{\widehat{m}}$. The remaining question pertains to the choice of the characteristic distance $\mathcal {H}$ to take for the integral. Möri and Lecampion (2022, 2023) have demonstrated that the behavior of the head dominates buoyant hydraulic fractures. We thus consider that the relevant length over which energy is dissipated to promote buoyant growth is the characteristic length of the head. When the fracture propagates in the $\widehat{M}$-regime, the corresponding dimensionless head length is given as $\ell _{\widehat{m}}^\textrm{head}\left( t_d\right) /\ell _\textrm{b}^{\text {2D}} = 1$. From this knowledge, we can derive the limit for immediate breakthrough according to Eq. (18) considering the higher distance of $d = 175$ m. The results are respectively given by $K_{{\text {Ic-2}}}/K_{{\text {Ic-1}}}\vert _{d = 175} \le 10.86$ for the weak formation and 2.89 for the strong formation, as reported in Table 1.

**Fig. 6:**
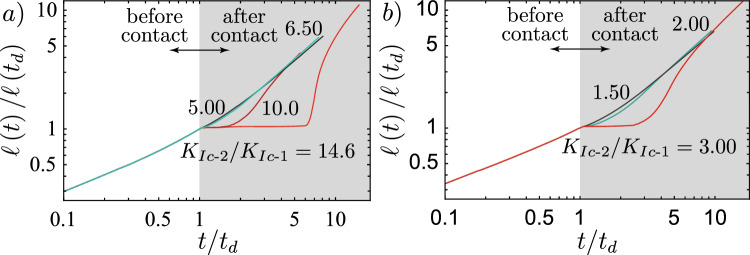
Testing of the limit for immediate breakthrough calculated using Eq. (18). **a** Simulations for the “weak” case of Table 1. Simulations are done for a toughness jump of 14.6 (red), 10.0 (dark red), 6.50 (light green), and 5.00 (brown). **b** Simulations for the “strong” case of Table 1 with $K_{{\text {Ic-2}}}/K_{{\text {Ic-1}}} = 3.00$ (red), 2.00 (light green), and 1.50 (brown). (Color figure online)

**Fig. 7:**
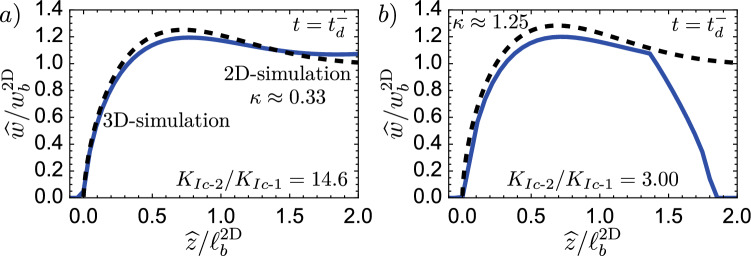
Comparison of the 2D opening profile (black-dashed line) with the opening profile obtained from the 3D simulation just before the fracture reaches the toughness jump. **a** Simulation for the “weak” case of Table 1 with a $K_{{\text {Ic-2}}}/K_{{\text {Ic-1}}} = 14.6$. **b** Simulations for the "strong" case of Table 1 with $K_{{\text {Ic-2}}}/K_{{\text {Ic-1}}} = 3.00$

We test these predictions through numerical simulations and present the results in Fig. 6. First, we perform simulations on the weak formation (see Fig. 6) and test with a value of $K_{{\text {Ic-2}}}/K_{{\text {Ic-1}}} = 14.6$, above the calculated limit of 10.86. The corresponding 3D simulation shows that this value, supposed to give an immediate breakthrough, leads to *transient containment*. We then gradually reduce the value of the jump to observe when we obtain an *immediate breakthrough*. The moment when the behavior changes from *transient containment* to *immediate breakthrough* is between 5.00 and 6.50. If we take a limiting value of 5.75, our prediction overestimates the necessary jump for an *immediate breakthrough* by approximately a factor of 2. To investigate why we have this overestimation, we compare in Fig. 7 the effective opening obtained to the 2D one. The first observation is that in both cases, up to the distance of $\widehat{z}/\ell _\textrm{b}^{\text {2D}}$, the actual opening is smaller than the 2D prediction. In Fig. 7b, we additionally see that the fracture has not yet grown up to the full size of the head. In addition to the opening, we calculate the actual velocity $V_\textrm{num} \approx 0.33$ m/s (scaling-based 0.43 m/s) from the time derivative of the fracture height growth and estimate the actual tail opening of the 3D simulation to obtain $Q_{2D} \approx 9.40 \times 10^{-4}$ m^2^/s (scaling-based $1.19 \times 10^{-3}$ m^2^/s). Taking these effective numerical values of the 3D simulations in the 2D estimate of Eq. (18) gives a slightly different limit for an *immediate breakthrough* of $K_{{\text {Ic-2}}}/K_{{\text {Ic-1}}}\vert _{d = 175, \textrm{num}} \le 9.56$. Even the numerically taken approximation along the centre line is still nearly double the observed value of about 5.75. We interpret this discrepancy to be related to a strong 3D effect. The same approximated 2D estimation works considerably better in the radial case presented in Peruzzo (2023). One reason for this is that the symmetry over the injection point allows them to match the opening of the semi-infinite fracture very well onto the radial, finite fracture. The situation is quite different for a non-axisymmetric configuration like the one presented in this contribution. Due to the finite extent parallel to the jump, local effects have a strong influence. Furthermore, we have neglected all pre-factors from previous numerical studies, and fewer semi- or analytical solutions are available. Nonetheless, the poor match, when compared to the numerically obtained opening, emphasizes that the difference stems mostly from the significant 3D effects. We can highlight this using the second parameter combination for the case of a strong rock formation. Albeit, the numerical limit obtained here is only slightly smaller than the scaling-based one $K_{{\text {Ic-2}}}/K_{{\text {Ic-1}}}\vert _{d = 175, \textrm{num}} \le 2.43$. For this set, Fig. 6 shows that the immediate breakthrough is observed for $K_{{\text {Ic-2}}}/K_{{\text {Ic-1}}} = 2.00$ and 1.50 but a transient containment exists already for $K_{{\text {Ic-2}}}/K_{{\text {Ic-1}}} = 3.00$. We would thus set the limit numerically at $K_{{\text {Ic-2}}}/K_{{\text {Ic-1}}} = 2.50$, which corresponds very well to the numerical 2D-prediction. Differences, in this case, are a mismatch of the velocities and that the fracture is still very close to its source point (the source point is at about $\widehat{z}/\ell _\textrm{b}^{\text {2D}} \approx 1.36$).

We use the simulations presented in Möri et al. (2023b) to validate further our estimations of the limits for an *immediate breakthrough*. Set number 1 of Möri et al. (2023b) encounters the toughness jump similarly in the $\widehat{M}$-regime. The calculated limit for immediate breakthrough in this case would be $K_{{\text {Ic-2}}}/K_{{\text {Ic-1}}} = 3.20$. Their simulations show that effectively the simulation with a small jump of $K_{{\text {Ic-2}}}/K_{{\text {Ic-1}}} = 2.00$ leads to an *immediate breakthrough*, and with a value of 5.00 the fracture becomes temporarily contained. No data points are available in between such that we cannot state how far their limit is from the simulation results. To further investigate this limit, we have performed different simulations that reach the jump in the $\widehat{M}$-regime. We notably ran simulations with dimensionless values of $\mathcal {M}_{\widehat{k}} = 1.00 \times 10^5$ (7), $\mathcal {B}_{ks} = 500$ (8), and $\mathcal {D} = 50$ (9) where we have a predicted limit for *immediate breakthrough* of $K_{{\text {Ic-2}}}/K_{{\text {Ic-1}}} = 11.18$. Our simulations show that the real value must lay somewhere between $K_{{\text {Ic-2}}}/K_{{\text {Ic-1}}} = 7.00$ and 14.00. We see the same tendency for a set of simulations with $\mathcal {M}_{\widehat{k}} = 1.00 \times 10^5$ (7), $\mathcal {B}_{ks} = 250$ (8), and $\mathcal {D} = 25$ (9). When applying the observation to the second parameter set of Möri et al. (2023b), we can investigate if our findings also work in a different regime upon the interaction between the fracture and the jump, in this case, the $\widehat{M}^{\left[ V\right] }$-regime. In this configuration, the dimensionless tip-toughness is no longer given by Eq. 19 and depends now on the dimensionless buoyancy $\mathcal {B}_{ks}$ (8) and the distance $\mathcal {D}$ (9) as $\kappa _{\widehat{m}}^{\left[ V\right] } = \mathcal {B}_{ks}^{-15/16}\mathcal {D}^{3/4}$. The limit one obtains in this case is given by $K_{{\text {Ic-2}}}/K_{{\text {Ic-1}}} = 5.39$. This value presents a significant overestimation, as already a value of $K_{{\text {Ic-2}}}/K_{{\text {Ic-1}}} = 5$ leads to an indefinite containment (Möri et al. 2023b). We get similar observations when looking at a toughness-dominated case with the interaction in the pulse buoyant regime ($\widehat{K}^{\left[ V\right] }$-regime). For a combination of $\mathcal {M}_{\widehat{k}} = 1.00$, $\mathcal {B}_{ks} = 1.25$, and $\mathcal {D} = 2.00$ we predict an *immediate breakthrough* for $K_{{\text {Ic-2}}}/K_{{\text {Ic-1}}} = 1.21$. We observe *immediate breakthroughs* up to jumps of $K_{{\text {Ic-2}}}/K_{{\text {Ic-1}}} = 1.125$. For larger values, a second mechanism becomes important. Changing the fracture toughness means changing the minimum volume required for buoyant propagation. We can relate the dimensionless buoyancy of the higher toughness to its counterpart in the injection layer as

20 $$\begin{aligned} \mathcal {B}_{ks\text {-}2} = \left( \frac{K_{{\text {Ic-2}}}}{K_{{\text {Ic-1}}}}\right) ^{-8/5} \mathcal {B}_{ks\text {-}1}. \end{aligned}$$

For the presented toughness-dominated case, values of $K_{{\text {Ic-2}}}/K_{{\text {Ic-1}}} \ge 1.15$ lead to a $\mathcal {B}_{ks\text {-}2} < 1$ and do thus not allow for any further buoyant propagation in the upper layer. This second additional condition is generally valid and would prevent growth in the upper layer.

From the observations in three different interaction regimes ($\widehat{M}$-, $\widehat{M}^{\left[ V\right] }$-, and $\widehat{K}^{\left[ V\right] }$-regime) we can say that the prediction of *immediate breakthrough* versus *containment* based on the energy balance of a 2D semi-infinite buoyant hydraulic fracture generally over-estimates the necessary jump. This means that a toughness jump seems more efficient in arresting buoyant hydraulic fractures, as predicted by this method. The numerical results obtained show that the absolute value of the necessary jump to avoid *immediate breakthrough* usually correspond to high, respectively very high, values of the fracture toughness (weak rock $K_{{\text {Ic-2}}} \approx 1.60$ MPa m^1/2^, strong rock $K_{{\text {Ic-2}}} \approx 5.00$ MPa m^1/2^).

### Limit of Indefinite Containment

The previous paragraph enlightens the limits of an *immediate breakthrough* without distinguishing if the obtained containment, if such is observed, is transient or indefinite. We look at Fig. 2 to study the potential indefinite containment. For both containment cases, one can identify that the spreading along the interface occurs at a slowly varying fracture height. A similar observation was made by Möri et al. (2023a) and is inherent to the assumption of PKN-fractures (Perkins and Kern 1961; Nordgren 1972). As discussed in the introduction, the problem of PKN-fractures has obtained recent interest by Dontsov (2022) and Peruzzo (2023). The two discuss the transition from a toughness- to a viscosity-dominated PKN regime and investigate the conditions for which the fracture can penetrate the higher toughness layer. We follow a mixture of the approaches presented in chapter 6 of Peruzzo (2023) and Dontsov (2022). Our evaluations assume that we can adopt a local 2D plane strain approximation within the spreading fracture with a linear pressure gradient, also known as Weertman’s pulse (Weertman 1971). Such a linear loading can be obtained by combining the buoyancy contrast’s gradient $\varDelta \gamma$ and a characteristic constant pressure $p_*$. In this approach, we hypothesize that the stress-intensity factor at the lower end of this fracture equals zero min$\left\{ K_\textrm{Ic}\right\} = 0$. This assumption allows us to relate the fracture height directly to $p_*$ and $\varDelta \gamma$ (see Eq. (37)).

**Fig. 8:**
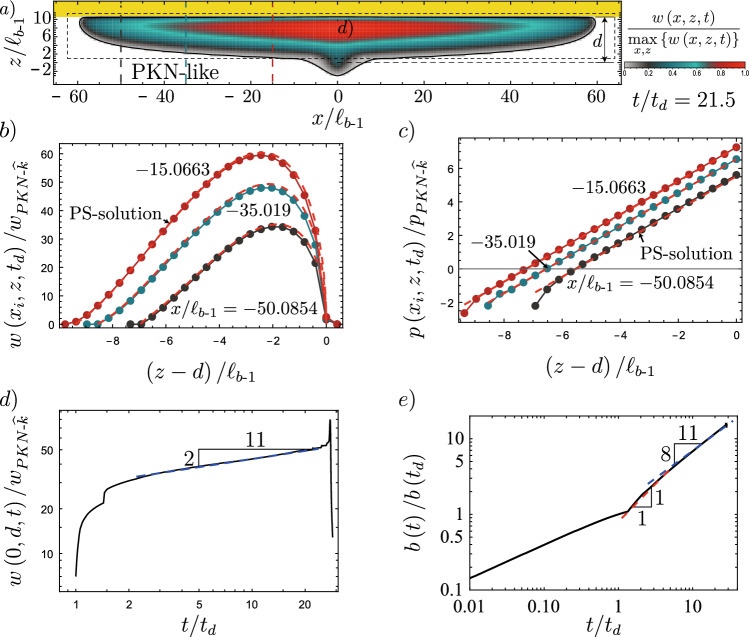
Extracted results from a simulation of the weak formation with $K_{{\text {Ic-2}}}/K_{{\text {Ic-1}}} = 18.65$ to compare the lateral spreading to the scaling-based results. **a** Footprint with opening distribution indicating the PKN-like area. Dashed vertical lines show where the opening (**b**) and pressure (**c**) profiles are extracted. **b** Opening profile (colored lines) with discretization (dots) and prediction according to the Weertman’s pulse (Weertman 1971) (PS-solution, red-dashed lines). **c** Pressure profiles with predicted pressure. **d** Evolution of the opening close to the breakthrough point (location indicated in **a**). The dashed line shows the tendency of the evolution in the PKN-$\widehat{M}$ regime. **e** Evolution of the maximum breadth, tracking the evolution of the $\ell _{PKN-*}$ with indicated expected power-laws of growth. For the PS-solutions of **b**, **c**, the values of $p_*$ and *h* are taken from the numerical simulations. (Color figure online)

We first investigate the toughness-dominated case. We assume that upward growth is not possible (this is the reason for the spreading). Downward growth is limited by min$\left\{ K_\textrm{Ic}\right\} = 0$ such that the fracture can only grow laterally. We use the local energy balance to calculate for which characteristic pressure $p_*$ lateral growth occurs. For the case of no fluid flow (purely toughness-dominated), it is possible to match the elastic energy stored in the 2D cross-section $U_{ps}$ with the energy release rate $G_{c\text {-}H}$ necessary to obtain lateral fracture growth

21 $$\begin{aligned} U_{ps}&= \frac{1}{2}\int _{2H} w\left( h, p_*\right) p\left( h, p_*\right) H\left( p_*\right) \text {d}h = 2H\frac{K_{{\text {Ic-1}}}^2}{E^{\prime }} = G_{c\text {-}H} \nonumber \\&\downarrow \\ p_{\text {PKN-}\widehat{k}}&= K_{{\text {Ic-1}}}^{2/3}\varDelta \gamma ^{1/3},\nonumber \end{aligned}$$

where $p\left( h, p_*\right)$ is the pressure given as a combination of $p_*$ and $\varDelta \gamma$. We observe that the obtained scale is equivalent to the pressure scale in the head of a toughness-dominated hydraulic fracture (Möri and Lecampion 2022, 2023). Using the global volume balance, we can directly obtain scales for the opening, fracture height, and length from the pressure scale. In the case of a continuous injection, we obtain

22 $$\begin{aligned} w_{\text {PKN-}\widehat{k}}&= \frac{K_{{\text {Ic-1}}}^{4/3}}{E^{\prime }\varDelta \gamma ^{1/3}}, \end{aligned}$$

23 $$\begin{aligned} p_{\text {PKN-}\widehat{k}}&= K_{{\text {Ic-1}}}^{2/3}\varDelta \gamma ^{1/3}, \end{aligned}$$

24 $$\begin{aligned} \ell _{\text {PKN-}\widehat{k}}&= \frac{E^{\prime }Q_\textrm{o}t\varDelta \gamma }{K_{{\text {Ic-1}}}^2}, \end{aligned}$$

25 $$\begin{aligned} h_{\text {PKN-}\widehat{k}}&= \ell _{b\text {-}1} = \left( \frac{K_{{\text {Ic-1}}}}{\varDelta \gamma }\right) ^{2/3}. \end{aligned}$$

Note that to obtain these scales, we have assumed that the volume entering the spreading fracture corresponds precisely to the volume injected, and we did not consider that lateral spreading only starts after the fracture reaches the interface at $t = t_d$. As pointed out by Möri and Lecampion (2021) and Peirce (2022), we can obtain the equivalent scale for a finite volume release by simply replacing $Q_\textrm{o}$ with $V_\textrm{o}/t$. We must evaluate the maximum value of the stress-intensity factor at the interface to evaluate if the fracture suffers from a breakthrough into the upper layer during this growth. As the pressure, height, and opening all along the fracture are constant, the stress intensity factor does not change. This value can be obtained from a Weertman’s pulse approximation (Weertman 1971) by propagating pre-factors as

26 $$\begin{aligned} \text {max}\left\{ K_\textrm{I}\right\} = \frac{4}{\sqrt{3}} K_{{\text {Ic-1}}}. \end{aligned}$$

This would mean that lateral propagation is energetically not favorable for any smaller change in toughness, and breakthrough would be nearly immediate. We have so far not gotten any simulations indicating a different behavior, as all simulations with a toughness-jump of $K_{{\text {Ic-2}}}/K_{{\text {Ic-1}}} < 4/\sqrt{3}$ (see for example the simulations shown in Möri et al. (2023b)) always lead to an *immediate breakthrough*, if in parallel the condition of Eq. (20) is satisfied. We also observe that if the fracture remains toughness-dominated, no breakthrough could ever happen if fracture containment is present initially.

However, the transition from the early time toughness- to the late time viscosity-dominated regime has recently been shown by Dontsov (2022); Peruzzo (2023), and Garagash (2023). As we consider a similar behavior for the PKN-like fracture along the interface, we follow the approach of Dontsov (2022) and approach the problem from a balance of momentum of the lateral flow, neglecting any contribution of fracture energy. With our base assumptions, we can obtain the average opening in the 2D section as a function of this $p_*$ and derive the expression of lateral flow in this PKN-like fracture. We then obtain an expression similar to equation (8) of Dontsov (2022) (see Eq. (40)) for a semi-infinite fracture propagating laterally. We apply the procedure outlined in Sect. 4 of Dontsov (2022) to obtain the relevant scales of lateral fracture growth along the interface.

27 $$\begin{aligned} w_{\text {PKN-}\widehat{m}}&= \frac{Q_\textrm{o}^{4/11}\varDelta \gamma ^{1/11}\mu ^{\prime 2/11}t^{2/11}}{E^{\prime 3/11}},\end{aligned}$$

28 $$\begin{aligned} p_{\text {PKN-}\widehat{m}}&= E^{\prime 4/11} Q_\textrm{o}^{2/11} \varDelta \gamma ^{6/11}\mu ^{\prime 1/11}t^{1/11}, \end{aligned}$$

29 $$\begin{aligned} \ell _{\text {PKN-}\widehat{m}}&= \frac{Q_\textrm{o}^{5/11}\varDelta \gamma ^{4/11}t^{8/11}}{E^{\prime 1/11}\mu ^{\prime 3/11}}, \end{aligned}$$

30 $$\begin{aligned} h_{\text {PKN-}\widehat{m}}&= \frac{E^{\prime 4/11}Q_\textrm{o}^{2/11}\mu ^{\prime 1/11}t^{1/11}}{\varDelta \gamma ^{5/11}}. \end{aligned}$$

In the process of obtaining these scales, we dropped pre-factors and abstain from explicitly solving for the evolution of pressure, opening, and height along the fracture (e.g., we do not solve for the functional $f\left( \xi \right)$ given in Dontsov (2022)). From the scales (27)–(30), we see that the fracture height and pressure increase with time. Consequently, the maximum stress intensity factor (at the interface) will also increase. We get the scale of the evolution of the stress intensity factor as

31 $$\begin{aligned} \text {max}\left\{ K_\textrm{I}\left( t\right) \right\} = E^{\prime 6/11}Q_\textrm{o}^{3/11}\varDelta \gamma ^{7/22}\mu ^{\prime 3/22}t^{3/22}. \end{aligned}$$

We expect breakthrough to occur once we have $\text {max}\left\{ K_\textrm{I}\left( t\right) \right\} = K_{{\text {Ic-2}}}$. By equalizing $K_{{\text {Ic-2}}}$ to Eq. (31) we can obtain the breakthrough time $t_\textrm{bt}$

32 $$\begin{aligned} t_\textrm{bt} = \frac{K_{{\text {Ic-2}}}^{22/3}}{E^{\prime 4}Q_\textrm{o}^2\varDelta \gamma ^{7/3}\mu ^{\prime }}. \end{aligned}$$

The implications of the scales (27)–(30) leading to the derivation of a breakthrough time is that breakthrough will **always** occur if the injection is not finite. The fracture necessarily goes from toughness- to viscosity-dominated propagation and will grow in this regime until a breakthrough occurs. This observation is not astonishing and has similarly been demonstrated by Peruzzo (2023) for the case without buoyancy effects. The only possibility to become *indefinitely contained* is thus given by the finite volume of the injection.

As in the radial case and for the PKN-$\widehat{K}$-regime, the finite volume scales of the PKN-$\widehat{M}$-regime can be obtained from the scales (27)-(30) by substituting $Q_\textrm{o}$ with $V_\textrm{o}/t$. We thus immediately observe that the opening, pressure, and height start to reduce with time. Consequently, the maximum stress intensity factor will also be reduced (see Appendix 2 for the details). In this regime, the fracture will transition from viscosity- to toughness-dominated, where it finally stops spreading laterally. A direct consequence of this reduction in stress-intensity factor is that breakthroughs can only happen during the injection, meaning that we can obtain the limiting volume necessary for the fracture to breakthrough for a given toughness jump by comparing the time of shut-in $t_\textrm{s}$ with the breakthrough time $t_\textrm{bt}$ (32)

33 $$\begin{aligned} \frac{t_\textrm{bt}}{t_\textrm{s}} = \frac{K_{{\text {Ic-2}}}^{22/3}}{E^{\prime 4}Q_\textrm{o}^2t_\textrm{s}\varDelta \gamma ^{7/3}\mu ^{\prime }} \le 1 \rightarrow \frac{K_{{\text {Ic-2}}}}{K_{{\text {Ic-1}}}} \le \mathcal {M}_{\widehat{k}}^{3/22}\mathcal {B}_{ks}^{5/22}. \end{aligned}$$

The inequality resulting from this comparison, right of the arrow in (33), gives the predicted limit for which a fracture can still break through.

**Fig. 9:**
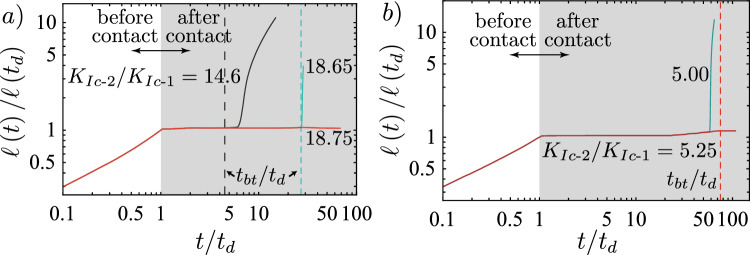
Testing of the limit for *indefinite containment* calculated using Eq. (33). **a** Simulations for the “weak” case of Table 1. Simulations are done for a toughness jump of 18.75 (red), 18.65 (light green), and 14.6 (brown). **b** Simulations for the “strong” case of Table 1 with $K_{{\text {Ic-2}}}/K_{{\text {Ic-1}}} = 5.25$ (red) and 5.00 (light green). (Color figure online)

#### Results for the Representative Cases

We now validate this limit again using the weak and strong rock parameter sets. The limits for the two cases are respectively given by $K_{{\text {Ic-2}}}/K_{{\text {Ic-1}}} = 18.7$ for the weak and $K_{{\text {Ic-2}}}/K_{{\text {Ic-1}}} = 4.97$ for the strong formation. Observation of Fig. 9a) shows that we effectively observe a *indefinite containment* for a toughness jump of $K_{{\text {Ic-2}}}/K_{{\text {Ic-1}}} = 18.75$ just above the limit and a breakthrough (*transient containment*) for the value just below ($K_{{\text {Ic-2}}}/K_{{\text {Ic-1}}} = 18.65$). In fact, Fig. 8b, c show the excellent agreement between the 2D plane-strain prediction of the Weertman’s pulse (Weertman 1971) with regard to the opening and pressure in the horizontal cross-section. In this comparison, we have matched the fracture height to the numerical results of the 3D simulation and taken the characteristic pressure $p_*$ from the numerical average over the fracture height. The pressure gradient thus matches $\varDelta \gamma$ perfectly, and the plane-strain assumption becomes valid. We further observe that the opening at the centre of the PKN-like spreading seems to follow the predicted power law very well (Fig. 8d). The same seems to hold for the evolution of the breadth *b*, representing the length $\ell _{PKN}$ of the PKN-like fracture. The initial phase closely resembles the predicted toughness-dominated behavior before the spreading tends to follow the viscosity-dominated prediction. Going back to Fig. 9a, the prediction of the breakthrough time works nearly perfectly for the case of $K_{{\text {Ic-2}}}/K_{{\text {Ic-1}}} = 18.65$ ($t_\textrm{bt} / t_\textrm{s}\vert _\textrm{num} \approx 0.974$ and $t_\textrm{bt} / t_\textrm{s}\vert _\textrm{scaling} = 0.972$) but presents a larger difference for the case when $K_{{\text {Ic-2}}}/K_{{\text {Ic-1}}} = 14.6$ ($t_\textrm{bt} / t_\textrm{s}\vert _\textrm{num} \approx 0.217$ and $t_\textrm{bt} / t_\textrm{s}\vert _\textrm{scaling} = 0.161$). There are several factors explaining this difference. The first and probably strongest effect stems from the limited lateral extent/aspect ratio at the moment of breakthrough for the simulation with the smaller toughness jump. Peruzzo (2023) has recently demonstrated through numerical simulations that the plane-strain assumption becomes more valid the higher the aspect ratio of the fracture becomes (theoretically shown by Hills et al. (1996)). It is also worth noting that the fracture, in this case, is still transitioning from the PKN-$\widehat{K}$ to the PKN-$\widehat{M}$ regime, further limiting the validity of the underlying assumptions. Finally, we can also note that we pick the breakthrough as the moment when the fracture at the centre accelerates significantly. The fracture does penetrate the higher toughness layer already before. The moment of this first penetration is difficult to grasp but might be more representative of the definition of breakthrough adopted. For the strong rock formation, the conclusions do not significantly differ. This time, we observe a breakthrough for a value of the jump slightly above the predicted limit $K_{{\text {Ic-2}}}/K_{{\text {Ic-1}}} = 5.00$ but still see the confinement for a value similarly greater than in the weak formation. In this case, we have an overestimation of the breakthrough time ($t_\textrm{bt} / t_\textrm{s}\vert _\textrm{num} \approx 0.814$ and $t_\textrm{bt} / t_\textrm{s}\vert _\textrm{scaling} = 1.051$). The overestimation is a direct consequence of the jump leading to the breakthrough being above the calculated limit. Necessarily, the fracture must break through before its predicted time, or else the breakthrough will not occur. The fact that the theory, despite a significant spreading, might not hold for this case is within the assumption of the injection history. We simplified the lateral spreading to a maximum and considered that the injection follows the same history and timing as at the injection point. Of course, a delay occurs between the injection start and when the fracture starts to spread laterally. Additionally, the initial injection rate will be non-uniform and will not decrease sharply to zero. Instead, fluid will flow from the tail at a decreasing rate into the spreading part. As such, the stress-intensity factor must not necessarily decrease immediately at shut-in and could continue to increase for a limited time. This ongoing increase might lead to a breakthrough after the predicted breakthrough time.

We recheck our theory for the two cases presented in Möri et al. (2023b). For their Set 1, the predicted limit would be at $K_{{\text {Ic-2}}}/K_{{\text {Ic-1}}} = 5.40$. Effectively, the simulation ran with $K_{{\text {Ic-2}}}/K_{{\text {Ic-1}}} = 5.00$ shows a transient containment where the predicted breakthrough time in this case is similar to the actual breakthrough time observed ($t_\textrm{bt} / t_\textrm{s}\vert _\textrm{num} \approx 0.567$ and $t_\textrm{bt} / t_\textrm{s}\vert _\textrm{scaling} = 0.875$). The study of their second case is somewhat different as the interaction occurs in the pulse $\widehat{M}^{\left[ V\right] }$-regime. The consequence is that the fracture either breaks through immediately or not at all. As soon as containment exists, we expect a decreasing stress intensity factor. Thus, no more breakthroughs are possible, indicating that Eq. (33) is only valid if the formation of a PKN-$\widehat{*}$ (constant height fracture with "high" aspect ratio) crack during the fluid release occurs. We use again our simulations with the different parameter-set leading to $\mathcal {M}_{\widehat{k}} = 1.00 \times 10^5$ (7), $\mathcal {B}_{ks} = 500$ (8), and $\mathcal {D} = 50$ (9). For these simulations, we observe lateral spreading during the injection and would predict with Eq. (33) a limiting value of $K_{{\text {Ic-2}}}/K_{{\text {Ic-1}}} = 19.7$. The numerically found value is close to this limit and is at approximately $K_{{\text {Ic-2}}}/K_{{\text {Ic-1}}} = 18.25$. The prediction of the breakthrough time for the corresponding transient containment cases is again within the order of magnitude and ($t_\textrm{bt} / t_\textrm{s}\vert _\textrm{num} \approx 1.115$ and $t_\textrm{bt} / t_\textrm{s}\vert _\textrm{scaling} = 0.509$ for $K_{{\text {Ic-2}}}/K_{{\text {Ic-1}}} = 18.00$ and $t_\textrm{bt} / t_\textrm{s}\vert _\textrm{num} \approx 0.873$ and $t_\textrm{bt} / t_\textrm{s}\vert _\textrm{pred} = 0.269$ for $K_{{\text {Ic-2}}}/K_{{\text {Ic-1}}} = 16.5$).

**Fig. 10:**
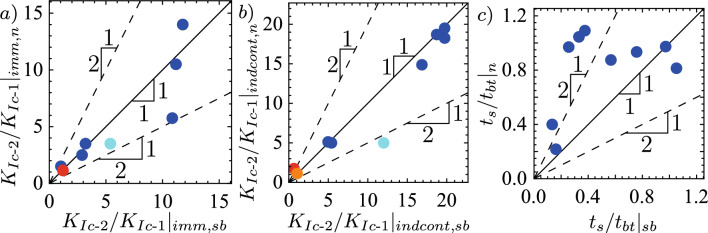
Overview of comparing the scaling-based predictions based on numerous simplifications compared to the results of full 3D simulations. Dots correspond to single evaluations where the color indicates the regime upon impact (blue—$\widehat{M}$, cyan—$\widehat{M}^{\left[ V\right] }$, red—$\widehat{K}$, orange—$\widehat{K}^{\left[ V\right] }$). **a** Predicted limit for *immediate breakthrough* (18) compared to results of numerical simulations. **b** Predicted limit for *indefinite containment* (33) compared to results of numerical simulations. **c** Predicted breakthrough time $t_\textrm{bt}$ (32) compared to the numerically observed breakthrough time. (Color figure online)

### Main Findings

We regroup the findings regarding the *immediate breakthrough* and the *indefinite containment* of buoyant hydraulic fractures reaching a change in fracture toughness in Fig. 10. Figure 10a compares the scaling-based predicted limit for *immediate breakthrough* based on the 2D, semi-infinite assumption compared to the effectively obtained numerical value from full 3D simulations. We use the mid-point between simulations where *immediate breakthrough* occurred and the closest value showing a fracture containment. The colors of the dots indicate the dominating regime upon the interaction with the toughness jump. In most cases, the scaling-based approach overestimates the obtained value. Nonetheless, the order of magnitude is well captured and most predictions are off by only about 30%. Some evaluations might however show discrepancies up to a factor of two regarding the prediction. Another observation is that for most evaluations, a value close to the maximum toughness jump observed in laboratory measurements of sedimentary rock beddings identified by Peruzzo (2023) of $K_{{\text {Ic-2}}}/K_{{\text {Ic-1}}} = 5.00$ leads to an immediate breakthrough and can hardly arrest a fracture. The exception is notably the toughness-dominated case where a value of $K_{{\text {Ic-2}}}/K_{{\text {Ic-1}}} \approx 1.14$ is already sufficient. The work of Möri and Lecampion (2022, 2023) has shown that this regime is difficult to obtain in real-world applications. Interestingly, the evaluation of the limit for *indefinite containment*, Fig. 10b, seems to be very well captured by the PKN-$\widehat{*}$ developments. The only exception here is the case where the fracture reaches the interface in the $\widehat{M}^{\left[ V\right] }$-regime. In this case, a lateral spreading according to the PKN-$\widehat{*}$ approach is, however, not possible, and the limit for *indefinite containment* is given by the limit for *immediate breakthrough*. Despite the good match in predicting the limiting value for *indefinite containment*, the prediction of the breakthrough time (32) seems to lead to a significant underestimation. However, we expect underestimation to diminish if we consider the differences in the injection history of the laterally growing fracture compared to the overall fracture injection history.

## Stress Barrier

The previous section has shown that substantial changes in stress-intensity factors are usually necessary to contain a fracture indefinitely, making it a very inefficient arrest mechanism. For radial fractures, the mechanism considered as most efficient are differences in the confining stress, so-called stress jumps (Harrison et al. 1954; Simonson et al. 1978; Adachi et al. 2010). For the case of buoyant hydraulic fractures, Möri et al. (2023b) have similarly hinted toward the high efficiency of such stress changes. For the cases they studied, stress jumps of $\varDelta \sigma \sim 1.00$ MPa were sufficient to arrest the fracture. The range of possible stress jumps can be identified to lay somewhere between 1.00 and 20.0 MPa (see e.g., (Haimson and Lee 1980; Leeman 1965; Adachi et al. 2007)). This indicates that stress jumps are more efficient and, in practice, the more realistic component leading to the arrest of buoyant hydraulic fractures. Möri et al. (2023b) proposed to non-dimensionalize the necessary stress jump using the characteristic pressure in the head of a toughness-dominated buoyant hydraulic fracture

34 $$\begin{aligned} \mathcal {S} = \frac{\varDelta \sigma }{p_{\widehat{k}}^\textrm{head}} = \frac{\varDelta \sigma }{K_\textrm{Ic}^{2/3}\varDelta \gamma ^{1/3}}. \end{aligned}$$

**Fig. 11:**
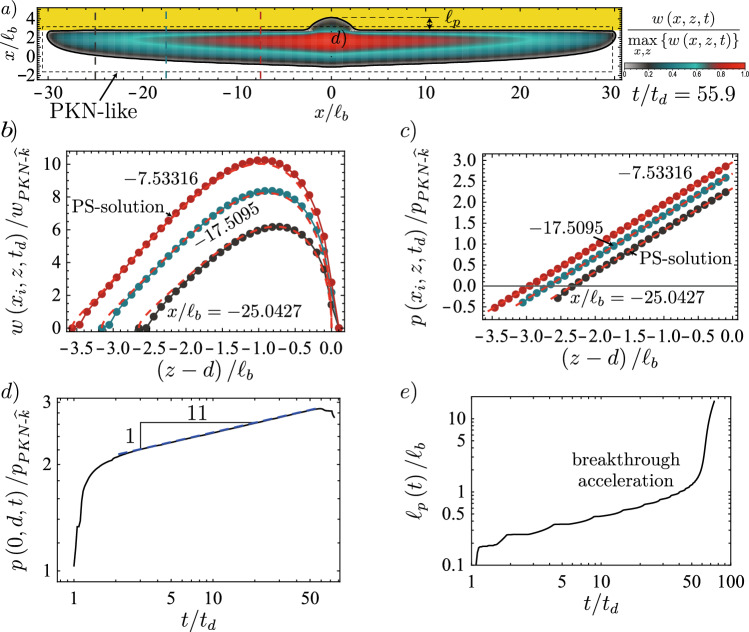
Extracted results from a simulation of the strong formation with $\mathcal {S} = 4.09$ (34) to compare the lateral spreading to the scaling approach. **a** Footprint with opening distribution indicating the PKN-like area. Dashed vertical lines show where the opening (**b**) and pressure (**c**) profiles are extracted. **b** Opening profile (colored lines) with discretization (dots) and prediction according to the 2D plane strain solution (PS-solution, red-dashed lines). **c** Pressure profiles with predicted pressure. **d** Evolution of the pressure close to the breakthrough point (location indicated in **a**). The dashed line shows the power law of the evolution in the PKN-$\widehat{M}$ regime. **e** Evolution of the penetration depth $\ell _p$ (defined in a) as the height of the fracture in the high-stress layer). For the PS-solutions of **b**, **c**, the values of $p_*$ and *h* are taken from the numerical simulations. (Color figure online)

We observe here that this is strictly equivalent to the characteristic pressure of a laterally spreading PKN-$\widehat{K}$ fracture. We further notice that the characteristic pressure in the head of a viscosity-dominated buoyant fracture can always be related to this characteristic pressure of the hydrostatically loaded 2D fracture. As this configuration has been shown to represent the limit for buoyant propagation in a given formation (Weertman 1971; Spence and Turcotte 1985; Lister and Kerr 1991; Möri and Lecampion 2023), we should be able to characterize the possibility of a stress barrier to arrest buoyant hydraulic fractures by comparing the value of the dimensionless stress jump to the characteristic pressure in the head of this fracture. With this analysis, we would investigate the possibility of the fracture to break through the barrier immediately. The terminology of possible outcomes for stress barriers differs from the toughness jump case. The fracture must penetrate the upper layer to "feel" the stress change. The fracture thus always breaks into the higher format. In the case of a stress barrier, we thus adopt a slightly different definition of *immediate breakthrough*, which is now defined by not having a significant acceleration phase in the higher stress layer (see the characteristic velocity change in Fig. 11e).

As discussed before, the two combinations of parameters presented in Table 1 lead to an interaction with the stress jump in the $\widehat{M}$-regime. According to Möri and Lecampion (2022), the head pressure in this regime is related to the characteristic pressure of a hydrostatically loaded fracture as

35 $$\begin{aligned} \frac{p_{\widehat{m}}^\textrm{head}\left( t_d\right) }{p_{\widehat{k}}^\textrm{head}} = \mathcal {M}_{\widehat{k}}^{3/20}\mathcal {D}^{-1/20} \rightarrow \text {breakthrough if: } \mathcal {S} \le \mathcal {M}_{\widehat{k}}^{3/20}\mathcal {D}^{-1/20} - 1. \end{aligned}$$

Note that the $-1$ in Eq. (35) is because the residual pressure in the upper layer must remain larger or equal to the necessary characteristic head pressure, which is the one of a hydrostatically loaded radial fracture (Möri and Lecampion 2023). Similar to the concept of the change in stress intensity factor developed in Eqs. (31) to (33), we can assume a PKN-like behavior in the spreading. For a validation of this assumption, see Fig. 11b–d. Instead of evaluating the stress intensity factor, we set the pressure scale to the stress change ($+$ the characteristic head pressure) to obtain the breakthrough time and limit for indefinite containment

36 $$\begin{aligned} t_{bt,\mathcal {S}} = \frac{K_\textrm{Ic}^{22/3}}{E^{\prime 4}Q_\textrm{o}^2\varDelta \gamma ^{7/3}\mu ^{\prime }} \left( \mathcal {S}^{11} + 1\right) \rightarrow \mathcal {S} \ge \left( \mathcal {M}_{\widehat{k}}\mathcal {B}_{ks}^{5/3} - 1\right) ^{1/11}. \end{aligned}$$

Note the similarities in the breakthrough time between Eqs. (32) and (36). The two only differ in their expression related to the property which changes. This is because the stress intensity factor depends directly on the pressure. As such, the two yield very similar results.

**Fig. 12:**
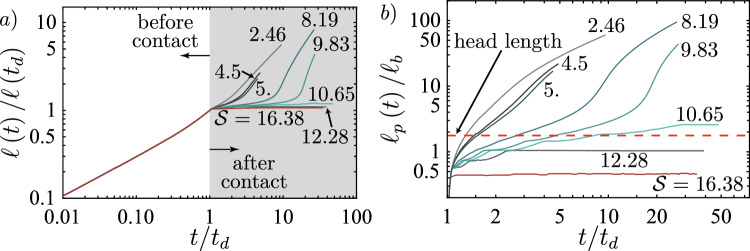
Numerical evaluation of the limits for *immediate breakthrough* (35) and *indefinite containment* (36) of the “weak” case of Table 1 for a given stress barrier $\mathcal {S}$ at a distance *d*. **a** Evolution of fracture height. Simulations are done for $\mathcal {S} = 16.38$, 12.28, 10.65, 9.83, 8.19, 5.00, 4.50, and 2.46 (red–green–gray). **b** Evolution of the penetration depth for the same simulations. (Color figure online)

We numerically check for these two estimations of limits using our strong and weak rock formations. The respective limits of *immediate breakthrough* are given as $\mathcal {S} \le 0.87$ (strong) and $\mathcal {S} \le 3.53$ (weak) and the ones for the *indefinite containment* as $\mathcal {S} \le 2.91$ (strong) and $\mathcal {S} \le 7.05$ (weak). Figure 12 shows the evaluation of the two limits for the weak formation. In Fig. 12b, we can see that no acceleration is observed for a value of $\mathcal {S} \le 2.46$ and starts to become more pronounced for $\mathcal {S} \le 4.50$, making the predicted limit fairly accurate. The limit for an *indefinite containment*, on the other hand, seems to be an underestimation of the necessary stress jump (numerics give $\mathcal {S} \approx 10$). It is important to note that the absolute value of the necessary stress jumps for indefinite containment is only about 0.625 MPa. This low value is way below the typical differences observed in the field. It also shows the enhanced capability in comparison to toughness jumps, as the value of the toughness jump necessary for *indefinite containment* of this case was $K_{{\text {Ic-2}}}/K_{{\text {Ic-1}}} \approx 18.70$. Similar observations hold for the strong rock formation. *Indefinite containment* is observed for a stress barrier with a magnitude of about $\mathcal {S} \approx 4.25$, such that the predicted value overestimates the actual limit. The evaluation of the *immediate breakthrough* shows that for a value of $\mathcal {S} \approx 1.64$ is already observed. This value is fairly close to the $\mathcal {S} \le 1.05$ prediction. For both limits and cases, we can thus see that the ad-hoc predictions based on the head pressure and pressure in a lateral PKN-like fracture are fairly accurate in predicting the interaction of a buoyant hydraulic fracture with a stress barrier. The absolute value of the stress jump necessary in this case is $\varDelta \sigma \sim 1$ MPa. This value is slightly higher than the limit required in the weak case but still at the lower end of actual stress jumps observed. It also aligns well with the observations of Möri et al. (2023b), which give the same order for the absolute value of the stress barrier. We can similarly adopt estimations for their second parameter set. For this case, we need to adapt the estimation for *immediate breakthrough* using the pressure evolution in the $\widehat{M}^{\left[ V\right] }$-regime. The limit for *immediate breakthrough* is thus given by $\mathcal {S} \le \mathcal {B}_{ks}^{5/8}\mathcal {D}^{-1/2} = 5.25$. This limit overestimates the necessary stress jump to limit *immediate breakthrough*. In Möri et al. (2023b) the limit is somewhere between $\varDelta \sigma = 0.50$ MPa and $\varDelta \sigma = 0.75$ MPa, which corresponds to $\mathcal {S}$ between 2.20 and 3.30. As the interaction is in a pulse regime, our theory does not allow for *transient containment*, so we do not have to calculate such a limit.

**Fig. 13:**
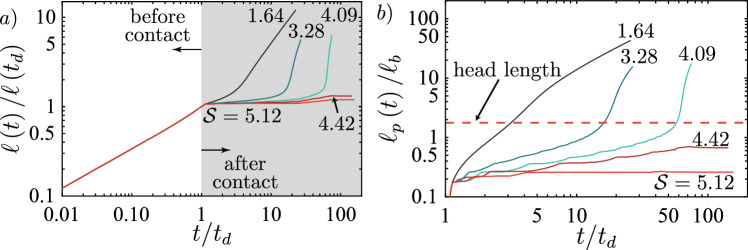
Numerical evaluation of the limits for *immediate breakthrough* (35) and *indefinite containment* (36) of the “weak” case of Table 1 for a given stress barrier $\mathcal {S}$ at a distance *d*. **a** Evolution of fracture height. Simulations are done for a $\mathcal {S} = 5.12$, 4.42, 4.09, 3.28, and 1.64 (red–green–gray). **b** Evolution of the penetration depth for the same simulations. (Color figure online)

This section shows that a simple prediction of the necessary magnitude of a stress barrier for the arrest of buoyant hydraulic fractures is possible using the same PKN-$\widehat{*}$ scaling to investigate the necessary toughness jump (see Sect. 3). It also demonstrates that stress barriers are highly efficient in arresting buoyant hydraulic fractures for representative values observed in the field (see e.g., (Haimson and Lee 1980; Leeman 1965; Adachi et al. 2007)) (Fig. 13).

## Discussion

We have investigated two possible mechanisms that could arrest buoyant hydraulic fractures: a jump in fracture toughness and stress. However, many other possible mechanisms of fracture arrest exist. We can notably list changes in the stress orientation, in elastic properties of the material (e.g., changes in *E* and $\nu$), fluid leak-off (i.e., rock permeability), and changes of the fracture toughness as a function of fracture velocity or fracture size. In other studies (Möri et al. 2023b; Möri 2023), the authors have already performed preliminary investigations of fluid leak-off and the role of a fracture size-dependent toughness. Hereafter, we will briefly discuss the effect of these two mechanisms and the combination of different arrest mechanisms.

### The Effect of Fluid Leak-Off

In industrial hydraulic fracturing applications, the most commonly adopted fluid leak-off model is Carter’s model (Carter 1957). For a discussion of the model’s validity, assumptions, and extensions to account for poroelasticity and pressure-dependent leak-off see, for example, Lecampion et al. (2018); Kovalyshen (2010); Kanin et al. (2020); Dontsov (2021); Gao and Detournay (2020, 2021). In the context of buoyant hydraulic fractures, the problem has obtained interest in volcanology thanks to the analogy between Carter’s leak-off model (Carter 1957) and the solidification of magmatic intrusions (Dontsov 2016). The analogy is based on the assumption of negligible advection in the fluid and a negligible excess temperature of the magma compared to the host rock. Bruce and Huppert (1990) applied these assumptions to obtain the velocity of the inward-moving solidification front, which has the same temporal dependence as Carter’s leak-off model. It is thus possible to model this solidification as a general loss of fluid, equivalently to the case of fluid leak-off (Turcotte and Schubert 2002; Bruce and Huppert 1990; Delaney and Pollard 1982; Petford et al. 1994; Rubin 1993). Another interesting study on semi-infinite buoyant hydraulic fractures has been performed by Dontsov (2016), who investigated the non-steady propagation of such fractures under a continuous release in 2D configurations. Möri (2023) observed a similar non-steady behavior for 3D planar buoyant hydraulic fractures for an ongoing fluid injection. These previous contributions also indicate that in the case of a finite volume injection, fluid leak-off will always arrest the buoyant propagation of hydraulic fractures. Möri et al. (2023b) performed several simulations on their representative cases, showing that moderate leak-off alone could already arrest the fracture. They also demonstrated that changes in the leak-off velocity are even more efficient in arresting fractures. As soon as a fracture is in a leak-off dominated regime, radial or buoyant, and the injection stops, the propagation of the fracture will come to a halt (Möri and Lecampion 2021; Peirce 2022; Peirce and Detournay 2022b). The fluid loss further reduces head volume, making the buoyant fracture more toughness-dominated. In toughness-dominated cases, smaller values of stress barriers and toughness jumps are usually required to arrest the fracture.

### The Role of a Fracture Size Dependent Toughness

In industrial applications (Rutledge et al. 2004; Mayerhofer et al. 2000; Garagash 2023) as in the context of magmatic intrusions (Delaney and Pollard 1981; Reches and Fink 1988; Pollard and Muller 1976) observations indicate that the energy required to fracture rock increases with the size of the fracture. In Möri et al. (2023b), the authors followed the approach of Liu et al. (2019) where the apparent fracture toughness is taken as a power law of the characteristic length scale of the fracture (see equation (1) of Möri et al. (2023b)). In the case of buoyant hydraulic fractures, Möri et al. (2023b) showed that this could only prevent the fracture from becoming buoyant but could not arrest an already buoyant fracture. They demonstrate that equivalent values of the dimensionless buoyancy $\mathcal {B}_{ks}$ (8) and the dimensionless viscosity $\mathcal {M}_{\widehat{k}}$ (7) which govern the problem as outlined in Sect. 2.2. Concerning the cases studied herein of the arrest of already buoyant fractures, the fracture size-dependent toughness has similar effects to fluid-leak-off in making fractures approaching the toughness-dominated regime. For toughness-dominated fractures, we repeat that the required changes in properties must be smaller to ultimately arrest the fracture.

### Combination of Arresting Mechanisms

We have studied and discussed several mechanisms capable of arresting buoyant hydraulic fractures separately. In almost all cases, multiple mechanisms will be present simultaneously. For example, changes in the fracture toughness $K_{{\text {Ic-2}}}/K_{{\text {Ic-1}}}$ will most likely be due to a lithology change. Such a lithology change will affect the toughness, elastic properties, and, most often, the confining stress. Furthermore, it affects the density of the solid, which will change the buoyancy, which we assumed constant herein. All these effects might favor containment or promote buoyant propagation (think, i.e., about a negative stress jump, which will accelerate the fracture). One main contributor to these considerations is fluid leak-off. Albeit potentially small, fluid leak-off is always present in industrial applications (as is solidification in volcanological considerations) and thus necessarily reduces the necessary strength of other mechanisms to arrest fractures potentially. From the individual analysis performed herein, we can already demonstrate that mechanisms like stress barriers are very efficient in arresting buoyant hydraulic fractures. If they are combined with leak-off and maybe a size-dependent toughness, the risk of industrially created hydraulic fractures traveling over significant vertical distances as buoyant fractures is extremely low. This differs from the observation of magmatic intrusion, which can also be modeled as hydraulic fractures. One possible explanation is that, when considering laboratory values for the fracture toughness, magmatic intrusions are more inclined to be in viscosity-dominated regimes (Davis et al. 2023; Möri and Lecampion 2023). Based on the observations on the higher capacity of such fractures to penetrate through heterogeneities, such a difference might partly explain the discrepancy between the frequently observed uprising of magmatic dykes and the fact that no records of industrial hydraulic fractures reaching the surface are known to date. It is, however, noteworthy that the actual value of the fracture toughness for large-scale magmatic intrusions is a big debate (see e.g., Heimpel and Olson (1994); Rivalta et al. (2015) and references therein). Furthermore, such fractures can be considered as having no leak-off thanks to the high fracturing fluid viscosity.

## Conclusions

We have studied the effect of increases in Mode I fracture toughness and stress barriers on fully developed buoyant hydraulic fractures. We note that our study does not consider any change in the elastic properties of the formation. We used the 3D planar hydraulic fracture solver PyFrac (Zia and Lecampion 2020) to validate scaling arguments and considered each mechanism separately. We distinguished between three possible interactions. *Immediate breakthrough*: The fracture does not significantly slow down at the location of the change in property/stress nor spread laterally along the interface of the change to a significant extent. *Transient containment*: The fracture stops (slows down significantly) its buoyant growth and spreads along the interface of the property/stress change before finally breaking through into the subsequent formation. *Indefinite containment*: The fracture stops its buoyant growth entirely and becomes arrested below (around) the location of the change in property/stress.

Our study shows that the derived simplified 2D development to estimate the necessary toughness jump for *immediate breakthrough* often overestimates the values observed in numerical evaluations. The approximate approach is based on reducing the planar 3D analysis to a 2D semi-infinite buoyant hydraulic fracture just before the contact with the interface of property changes. Despite the good agreement in the cross-sectional opening profile (see Fig. 7), the prediction for *immediate breakthrough* is off by nearly a factor of two in some cases (see Fig. 10a). We interpret this to be intrinsically linked to the 3D effects at play in this configuration. Our second development considered that the lateral spreading along the interface of fracture toughness changes can be approximated as a constant-height fracture and showed, in most cases studied herein, good agreement with the full 3D planar simulations (see Fig. 10b). As a result, it is possible to predict the necessary toughness jump for an *indefinite containment* as $K_{{\text {Ic-2}}}/K_{{\text {Ic-1}}} > \mathcal {M}_{\widehat{k}}^{3/22}\mathcal {B}_{ks}^{5/22}$ (33). On the other hand, the timing of the predicted breakthrough, in the case of a *transient containment*, shows similar errors to the estimation given for *immediate breakthrough* (see Fig. 10c). The reason is that the timing depends much more on the injection history into the constant-height fracture than the breakthrough itself (see similar observations in Möri and Lecampion (2021, 2023)). To get a better estimation of this phenomenon, more advanced considerations on the lateral spreading would be necessary.

In the case of a stress barrier, the observations from our numerical simulations compared to scaling developments to predict the corresponding limits are similar. Like in the case of a toughness jump, the 2D simplification fails, whereas the constant-height approximation provides an accurate estimate of the limiting value necessary for *indefinite containment* $\varDelta \sigma /\left( K_\textrm{Ic}^{2/3}\varDelta \gamma ^{1/3}\right) > \left( \mathcal {M}_{\widehat{k}}\mathcal {B}_{ks}^{5/3} - 1\right) ^{1/11}$ (36). Again, a refinement of the theoretical considerations would be possible, but the first-order effect is very well captured.

Our numerical evaluation also shows that the necessary toughness changes to arrest buoyant hydraulic fractures are usually higher than what is commonly observed in nature. On the other hand, stress barriers on the order of 1 MPa are most often sufficient to arrest buoyant hydraulic fractures. Such small values for stress variations are at the lower end of positive stress jumps observed in industrially stimulated reservoirs. In conclusion, they are likely one of the reasons why buoyant hydraulic fractures are not observed in industrial applications. Finally, combining these arrest mechanisms with others, like fluid leak-off, changes in the mechanical properties, a change of buoyancy, and more, explains why anthropogenic hydraulic fractures do not propagate over significant vertical distances upon cessation of the injection.

## Data Availability

Not applicable.

## Appendix 1. Dimensionless Tip-Toughness in Various Regimes

In Sect. 3.1, we have shown how the limit of immediate breakthrough can be related to the propagation of a 2D plane strain steadily moving semi-infinite hydraulic fracture. This type of fracture has been shown to depend on a dimensionless toughness given by Eq. (16) (Roper and Lister 2005, 2007), which can be related to the dimensionless numbers (7)–(9) as a function of the propagation regime of the buoyant hydraulic fracture. We present the corresponding tip-toughnesses in all regimes in Table 2 (see as an example (19)). We also define the dimensionless head length in the 2D semi-infinite scaling $\ell _*^\textrm{head}/\ell _\textrm{b}^{\text {2D}}$ (15)

**Table 2:** Dimensionless tip-toughness and head length as a function of the dominating regime of the buoyant hydraulic fracture

Dominating regime	$\widehat{K}$	$\widehat{K}^{\left[ V\right] }$	$\widehat{M}$	$\widehat{M}^\textrm{stabilized}$	$\widehat{M}^{\left[ V\right] }$
$\kappa _*$	$\mathcal {M}_{\widehat{k}}^{-1/4}$	$\mathcal {D}^{3/4}\mathcal {B}_{ks}^{-5/4}$	$\mathcal {D}^{3/40}\mathcal {M}_{\widehat{k}}^{-9/40}$	$\mathcal {M}_{\widehat{k}}^{-3/20}$	$\mathcal {D}^{3/4}\mathcal {B}_{ks}^{-15/16}$
$\ell _*^\textrm{head}/\ell _\textrm{b}^{\text {2D}}$	$\mathcal {M}_{\widehat{k}}^{-1/6}$	$\mathcal {D}^{1/2}\mathcal {B}_{ks}^{-5/6}$	1	1	1

## Appendix 2. Buoyant PKN-Like Viscosity-Dominated Scaling

Weertman (1971) solves for the possible propagation of a finite length, 2D, plane strain loaded by combining a constant pressure and a linear pressure gradient. The solution is based on fixing the maximum stress intensity factor at one end to the Mode I fracture toughness and the minimum stress intensity factor to zero. In this way, the fracture could propagate at the maximum stress-intensity factor because it would close where the minimum stress-intensity factor is. For the case presented herein, we consider a 2D cross-section of the buoyant fracture spreading along the interface. We assume that the PKN-like (Perkins and Kern 1961; Nordgren 1972) does not penetrate the layer with the changed property. In the context of a change in Mode I fracture toughness, we have to slightly adapt the approach of Weertman (1971) as we do not know the value of the maximum-stress intensity factor (it is somewhere between $K_{{\text {Ic-1}}}$ and $K_{{\text {Ic-2}}}$). Setting the minimum stress intensity factor to zero relates the fracture half height *h* to the average pressure $p_*$ and the gradient $\varDelta \gamma$

37 $$\begin{aligned} \text {min}\left\{ K_\textrm{Ic}\right\} = \sqrt{\pi h}\left( p_*\left( x\right) - \frac{1}{2}\varDelta \gamma h\right) \rightarrow h = \frac{2p_*\left( x\right) }{\varDelta \gamma }, \end{aligned}$$

we assume that the buoyancy $\varDelta \gamma$ gives the linear pressure gradient. We can then obtain the average opening in the cross-section by elastic superposition and integration as

38 $$\begin{aligned} \overline{w} = 2\pi \frac{p_*\left( x\right) ^2}{E^{\prime }\varDelta \gamma}, \end{aligned}$$

from which we can derive the lateral flow along the interface.

39 $$\begin{aligned} q_x = -144\pi \frac{p_*\left( x\right) ^6}{E^{\prime 3}\varDelta \gamma ^3\mu ^{\prime }}\frac{\partial p_*\left( x\right) }{\partial x}. \end{aligned}$$

Following now the approach of Dontsov (2022), we establish the lateral volume balance of the flow in a semi-infinite framework to get

40 $$\begin{aligned} 2\pi \frac{V}{E^{\prime }\varDelta \gamma }p_*\left( x\right) ^{2} - \frac{144\pi }{7}\frac{1}{E^{\prime 3}\varDelta \gamma ^3\mu ^{\prime }}\frac{\partial p_*\left( x\right) ^7}{\partial x} = 0. \end{aligned}$$

If we solve Eq. (40) under the boundary condition that pressure at the tip is zero, we can obtain the pressure in function of the coordinate *x* (dropping pre-factors)

41 $$\begin{aligned} p_*\left( x\right) = V^{1/5}E^{\prime 2/5}\varDelta \gamma ^{2/5}\mu ^{\prime 1/5}x^{1/5}. \end{aligned}$$

Replacing the coordinate *x* with a scaled coordinate $\xi = x/\ell \left( t\right) _{PKN-*}$ and introduce the substitution of $x = \left( 1-\xi \right) f\left( \xi \right)$. After this substitution we replace the definition of $p_*\left( x\right)$ (41) in Eqs. (37) and (38) to solve the volume balance

42 $$\begin{aligned} Q_\textrm{o} t \sim 2 h w l \rightarrow \ell _{\text {PKN-}\widehat{m}} \sim \frac{Q_\textrm{o}^{5/11}\varDelta \gamma ^{4/11}}{E^{\prime 1/11}\mu ^{\prime 3/11}}t^{8/11}, \end{aligned}$$

where we have dropped all pre-factors and do not explicitly solve for the function $f\left( \xi \right)$. Trough back substitution into Eqs. (37), (38), and (41), we obtain the scales (30) to (27).

Finally, we can obtain the maximum stress-intensity factor in the PKN fracture as

43 $$\begin{aligned}{} & {} \text {max}\left\{ K_\textrm{Ic}\right\} = \sqrt{\pi h}\left( p_*\left( x\right) + \frac{1}{2}\varDelta \gamma h\right) \rightarrow K_\textrm{Ic}\left( x, t\right) \nonumber \\{} & {} \quad = E^{\prime 6/11} Q_\textrm{o}^{3/11} \varDelta \gamma ^{7/22}\mu ^{\prime 3/22}t^{3/22}. \end{aligned}$$

from which we can derive the breakthrough time given in Eq. (32).

We present all scales of the lateral PKN scaling and associated transition times in Tables 3 and 4.

**Table 3:** Scales of the laterally spreading buoyant PKN fracture

	Constant injection		Finite volume	
	PKN-$\widehat{K}$	PKN-$\widehat{M}$	PKN-$\widehat{K}^{\left[ V\right] }$	PKN-$\widehat{M}^{\left[ V\right] }$
$\ell _{\text {PKN-}*} \vert \ell _{\text {PKN-}*}^{\left[ V\right] }$	$\frac{E^{\prime }Q_\textrm{o}\varDelta \gamma t}{K_\textrm{Ic}^2}$	$\frac{Q_\textrm{o}^{5/11}\varDelta \gamma ^{4/11}t^{8/11}}{E^{\prime 1/11}\mu ^{\prime 3/11}}$	$\frac{E^{\prime }V_\textrm{o}\varDelta \gamma }{K_\textrm{Ic}^2}$	$\frac{V_\textrm{o}^{5/11}\varDelta \gamma ^{4/11}t^{3/11}}{E^{\prime 1/11}\mu ^{\prime 3/11}}$
$h_{\text {PKN-}*} \vert h_{\text {PKN-}*}^{\left[ V\right] }$	$\ell _\textrm{b} = \frac{K_\textrm{Ic}^{2/3}}{\varDelta \gamma ^{2/3}}$	$\frac{E^{\prime 4/11}\mu ^{\prime 1/11}Q_\textrm{o}^{2/11}t^{1/11}}{\varDelta \gamma ^{5/11}}$	$\ell _\textrm{b}$	$\frac{E^{\prime 4/11}\mu ^{\prime 1/11}V_\textrm{o}^{2/11}}{\varDelta \gamma ^{5/11}t^{1/11}}$
$w_{\text {PKN-}*} \vert w_{\text {PKN-}*}^{\left[ V\right] }$	$\frac{K_\textrm{Ic}^{4/3}}{E^{\prime }\varDelta \gamma ^{1/3}}$	$\frac{\mu ^{\prime 2/11}\varDelta \gamma ^{1/11}Q_\textrm{o}^{4/11}t^{2/11}}{E^{\prime 3/11}}$	$\frac{K_\textrm{Ic}^{4/3}}{E^{\prime }\varDelta \gamma ^{1/3}}$	$\frac{\mu ^{\prime 2/11}\varDelta \gamma ^{1/11}V_\textrm{o}^{4/11}}{E^{\prime 3/11}t^{2/11}}$
$p_{\text {PKN-}*} \vert p_{\text {PKN-}*}^{\left[ V\right] }$	$K_\textrm{Ic}^{2/3}\varDelta \gamma ^{1/3}$	$E^{\prime 4/11}\varDelta \gamma ^{6/11}\mu ^{\prime 1/11}Q_\textrm{o}^{2/11}t^{1/11}$	$K_\textrm{Ic}^{2/3}\varDelta \gamma ^{1/3}$	$\frac{E^{\prime 4/11}\varDelta \gamma ^{6/11}\mu ^{\prime 1/11}V_\textrm{o}^{2/11}}{t^{1/11}}$
$\mathcal {M}_{\text {PKN-}*} \vert \mathcal {M}_{\text {PKN-}*}^{\left[ V\right] }$	$\mu ^{\prime } \frac{E^{\prime 4} \varDelta \gamma ^{7/3} Q_\textrm{o}^2 t}{K_\textrm{Ic}^{22/3}}$	1	$\mu ^{\prime } \frac{E^{\prime 4} \varDelta \gamma ^{7/3} V_\textrm{o}^2}{K_\textrm{Ic}^{22/3} t}$	1
$\mathcal {K}_{\text {PKN-}*} \vert \mathcal {K}_{\text {PKN-}*}^{\left[ V\right] }$	1	$K_\textrm{Ic} \frac{1}{E^{\prime 6/11}\varDelta \gamma ^{7/22}\mu ^{\prime 3/22} Q_\textrm{o}^{3/11} t^{3/22}}$	1	$K_\textrm{Ic} \frac{t^{3/22}}{E^{\prime 6/11}\varDelta \gamma ^{7/22}\mu ^{\prime 3/22} V_\textrm{o}^{3/11}}$

**Table 4:** Transition scales of buoyant PKN-like fractures in the continuous injection and finite volume scale

	$t_{\text {PKN-KM}} \vert t_{\text {PKN-MK}}^{\left[ V\right] }$	$\ell _{\text {PKN-KM}} \vert \ell _{\text {PKN-MK}}^{\left[ V\right] }$	$h_{\text {PKN-KM}} \vert h_{\text {PKN-MK}}^{\left[ V\right] }$	$w_{\text {PKN-KM}} \vert w_{\text {PKN-MK}}^{\left[ V\right] }$	$p_{\text {PKN-KM}} \vert p_{\text {PKN-MK}}^{\left[ V\right] }$
PKN-$\widehat{K}$ - PKN-$\widehat{M}$	$\frac{K_\textrm{Ic}^{22/3}}{E^{\prime 4}\varDelta \gamma ^{7/3}Q_\textrm{o}^2\mu ^{\prime }}$	$\frac{K_\textrm{Ic}^{16/3}}{E^{\prime 3}\varDelta \gamma ^{4/3}Q_\textrm{o}\mu ^{\prime }}$	$\ell _\textrm{b} = \frac{K_\textrm{Ic}^{2/3}}{\varDelta \gamma ^{2/3}}$	$\frac{K_\textrm{Ic}^{4/3}}{E^{\prime }\varDelta \gamma ^{1/3}}$	$K_\textrm{Ic}^{2/3}\varDelta \gamma ^{1/3}$
PKN-$\widehat{M}^{\left[ V\right] }$ - PKN-$\widehat{K}^{\left[ V\right] }$	$\frac{E^{\prime 4}\varDelta \gamma ^{7/3}V_\textrm{o}^2\mu ^{\prime }}{K_\textrm{Ic}^{22/3}}$	$\frac{E^{\prime } \varDelta \gamma V_\textrm{o}}{K_\textrm{Ic}^2}$	$\ell _\textrm{b} = \frac{K_\textrm{Ic}^{2/3}}{\varDelta \gamma ^{2/3}}$	$\frac{K_\textrm{Ic}^{4/3}}{E^{\prime }\varDelta \gamma ^{1/3}}$	$K_\textrm{Ic}^{2/3}\varDelta \gamma ^{1/3}$

## Code Availability

The data supporting this study’s findings are openly available at 10.5281/zenodo.10934293.
